# Exploring the role of β2-microglobulin in the relationship between physical activity and DNAm-predicted PhenoAge: Evidence from a population-based and mice single-cell RNA-sequencing study

**DOI:** 10.1016/j.jare.2025.11.047

**Published:** 2025-11-22

**Authors:** Yanwei You, Jinwei Li, Yang Zhang, Qiyu Liu, Alimjan Ablitip, Yongjie Lao, Jingtong Wang, Kailin Xu, Zhengbang Yao, Yuquan Chen, Xindong Ma

**Affiliations:** a Division of Sports Science & Physical Education, Tsinghua University, Beijing 100084, China; b IDG/McGovern Institute for Brain Research, Tsinghua University, Beijing 100084, China; c Saw Swee Hock School of Public Health, National University of Singapore, Singapore 117549, Singapore; d Department of Neurosurgery, West China Hospital, Sichuan University, Chengdu 610041, China; e Department of Vascular Surgery, Affiliated Cardiovascular Hospital of Kunming Medical University, Kunming 650500, China; f School of Life Sciences, Tsinghua University, Beijing 100084, China; g Hinda and Arthur Marcus Institute for Aging Research, Hebrew SeniorLife, Harvard Medical School, Boston, MA 02115, USA; h Department of Education Research, Centre for Addiction and Mental Health, Toronto, Ontario M6J 1H1, Canada; i School of Public Health and Preventive Medicine, Faculty of Medicine, Nursing & Health Sciences, Monash University, Victoria 3004, Australia

**Keywords:** β2-microglobulin, Physical activity, DNA methylation, PhenoAge, Population study, Mice study, Single-cell RNA sequencing

## Abstract

**Introduction:**

Physical activity (PA) is recognized as a cornerstone of healthy aging, yet the molecular mechanisms linking PA to biological aging remain poorly understood. β2-microglobulin (β2M), an inflammatory and aging biomarker, has emerged as a potential mediator of these effects. DNA methylation (DNAm)-based biological aging indicators, such as PhenoAge, provide a means to assess the relationship between PA, β2M, and aging at the molecular level.

**Objectives:**

This study aimed to investigate whether β2M mediates the association between PA and DNAm-predicted PhenoAge. Additionally, this study sought to explore the underlying molecular mechanisms using single-cell RNA sequencing (scRNA-seq) in mice.

**Methods:**

This study analyzed data from 936 participants in the U.S. population, assessing associations between PA, β2M levels, and PhenoAge using weighted multivariable regression and mediation models. β2M levels and PhenoAge were measured in blood samples and calculated using validated DNA methylation algorithms. To investigate molecular mechanisms, scRNA-seq was performed on peripheral blood samples from exercise and control mice.

**Results:**

In fully adjusted models, higher PA levels were significantly associated with lower PhenoAge (β = −0.014, p = 0.034) and β2M levels (β = −0.006, p = 0.032). Mediation analysis revealed that β2M mediated 37.67 % of the association between PA and PhenoAge (p = 0.042). Stratified analyses showed stronger effects in males and individuals with higher body mass index (BMI). In mice, scRNA-seq analysis demonstrated that exercise modulated β2M expression and enhanced immune, inflammatory, mitochondrial, and circadian pathways, particularly in B cells and myeloid cells.

**Conclusion:**

This study provides evidence that β2M mediates the beneficial effects of PA on biological aging. PA promotes healthy aging through molecular and cellular mechanisms, particularly benefiting individuals with higher baseline inflammation or metabolic dysfunction. These insights advance our understanding of the interplay between PA, β2M, and aging, offering directions for interventions to promote longevity and healthspan.

## Highlights

• Physical activity reduces DNAm-predicted PhenoAge via β2M mediation.
• β2M mediates 37.67 % of the association between physical activity and aging.
• Mice scRNA-seq reveals exercise modulates immune and mitochondrial pathways.
• β2M expression increases in B cells and myeloid cells with physical activity.
• Findings highlight β2M as a therapeutic target to promote healthy aging.

## Graphical abstract

**Figure:**
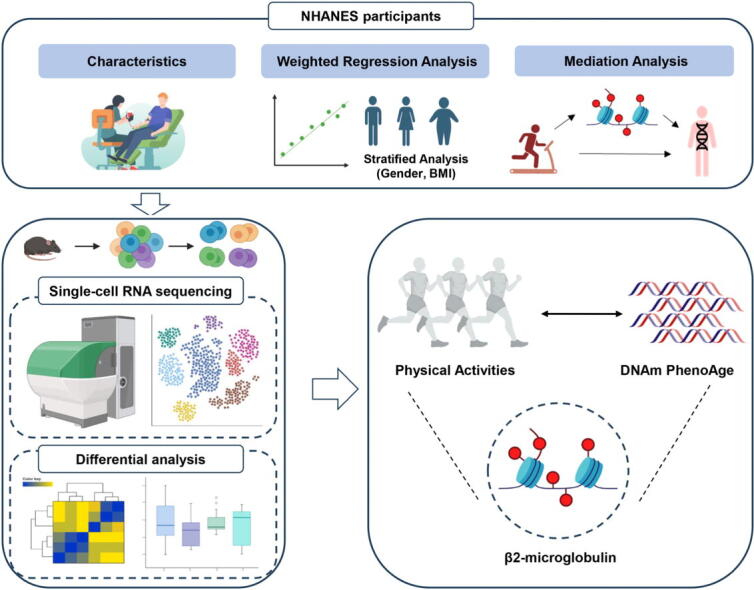

## Introduction

Aging is a process that involves the cross-talk of genetic, environmental, and lifestyle factors. Among lifestyle factors, physical activity (PA) has been shown to delay the onset of age-related diseases and improve overall health span [1,2]. Mechanistically, PA exerts its benefits by reducing chronic inflammation [3], oxidative stress [4], and cellular senescence [5]—key drivers of aging. DNA methylation (DNAm), an epigenetic modification that influences gene expression, has recently emerged as a powerful biomarker of biological aging [6]. DNAm biomarkers, including phenotypic age (PhenoAge) [7] and GrimAge [8], have been developed as predictors of biological aging, providing more accurate measures than chronological age. PhenoAge, in particular, is associated with various health outcomes such as all-cause mortality, cardiovascular disease, and cognitive decline, making it a critical measure of aging-related morbidity [9]. These markers capture the cumulative effects of environmental and lifestyle factors on the epigenome, offering a method for assessing biological aging.

Currently, evidence from both objectively measured physical activity using accelerometers and self-reported physical activity assessed through questionnaires shows a consistent relationship: engagement of physical activity may be associated with a lower PhenoAge [[10], [11], [12]]. Our previous investigation also found that PA may influence DNAm patterns linked to aging [[13], [14], [15]]. Despite advances in epigenetic research, the association between PA and these aging markers remains underexplored, particularly in diverse populations. Several studies have reported inverse associations between PA and DNAm of individual age-related genes like TP53, IL-6, NF-κB, and CXCL8 [16,17], suggesting that PA may contribute to slowing biological aging. However, comprehensive analyses that account for a range of covariates, such as gender, BMI, and socioeconomic status, are limited [18]. Moreover, whether PA influences the broader DNAm-predicted aging markers across different demographic groups, and how this relationship varies with factors such as gender or body weight, requires further investigation.

β2-microglobulin (β2M) is the soluble light chain of major histocompatibility complex class I (MHC-I) molecules [19,20], with elevated levels detected in the plasma and cerebrospinal fluid of aged mice and older adults [21]. Compared to β-amyloid, which is widely recognized for its pivotal role in aging and the pathogenesis of Alzheimer’s disease [22,23], β2M appears to be emerging as a novel biomarker in the context of aging and cognitive decline. Recent evidence suggests that β2M may represent a distinct and complementary mechanism underlying age-related neurodegeneration [24,25]. Unlike β-amyloid, which aggregates into plaques, β2M is a soluble protein linked to immune and inflammatory processes, with elevated levels observed in aging and certain neurodegenerative conditions [26]. Moreover, elevated circulating levels of β2M are associated with inflammation, immune dysfunction, and age-related decline in physical function [27,28]. However, whether β2M acts as a mediator of PA’s effects on PhenoAge remains unclear, warranting further investigation.

Notably, population-level observations often fail to provide direct insights into cellular and molecular mechanisms, as they are limited by confounding factors, heterogeneity across individuals, and the lack of experimental control. Animal models, particularly mice, offer a controlled environment to dissect the cellular and molecular mechanisms underlying aging. Single-cell RNA sequencing (scRNA-seq) has become a transformative tool in aging research [29], enabling the identification of cell-type-specific responses to PA and the characterization of aging-related molecular pathways. By integrating scRNA-seq with traditional measures of DNAm aging, it is possible to uncover how β2M expression is regulated across different cell types and how these changes relate to PA-induced benefits. Immunosenescence, the progressive deterioration of the immune system with age, has been linked to various age-related diseases [30,31], and PA is believed to mitigate these effects by modulating immune function. Studies leveraging scRNA-seq have demonstrated that PA can enhance the function of immune cells such as T cells and natural killer cells (NK cells) [32,33], potentially counteracting age-related immune decline. However, the integration of scRNA-seq data with epigenetic aging markers like DNAm-predicted PhenoAge is still in its infancy, and the role of PA in influencing these relationships warrants further exploration.

**Fig. 1:**
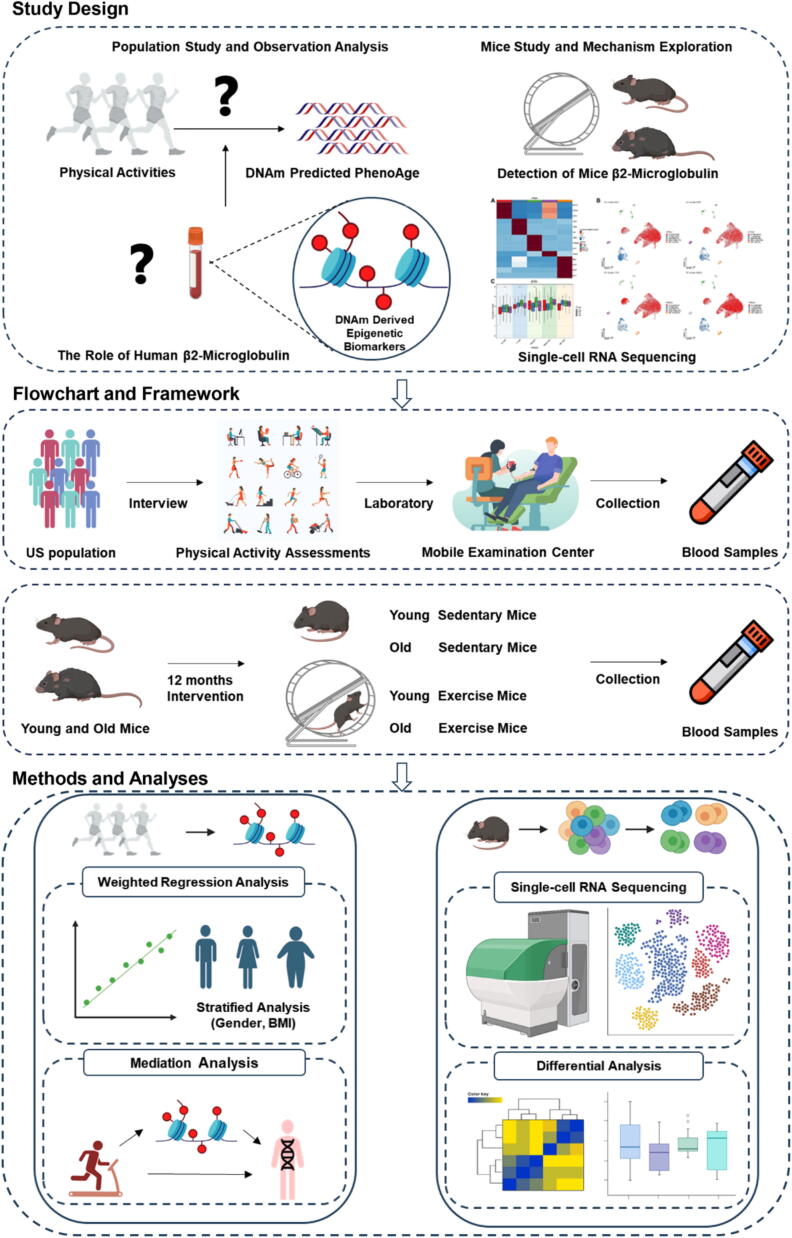
The Roadmap of This Study. Notes: At the top, the study design combines a population-based and a mouse-based study to investigate the role of β2M in the relationship between PA and DNAm-predicted PhenoAge. The population part examines associations between PA, β2M, and PhenoAge, while the mice part explores β2M expression and pathway activation using scRNA-seq. The middle section illustrates the flowchart and framework, detailing the human and animal studies. In the human part, data were collected from U.S. participants through interviews, PA assessments, laboratory tests, and blood sample collection at mobile examination centers. In the mice part, young and old mice underwent a 12-month intervention, divided into sedentary and exercise groups, followed by blood sample collection. The bottom section highlights the methods and analyses used. For the human part, weighted regression analysis was performed to assess the associations between PA, β2M, and PhenoAge, along with stratified analysis by gender and BMI and mediation analysis to examine the role of β2M as a mediator. For the mice part, scRNA-seq was applied to analyze β2M expression and perform differential pathway analysis.

This study aims to investigate the role of β2M in the relationship between PA and DNAm-prediced PhenoAge (Fig. 1). By combining blood samples from both population-based dataset and mice scRNA-seq, we seek to (1) assess the association between PA, β2M, and DNAm aging in human cohorts, (2) identify potential mediation relationship linking β2M, DNAm, and PhenoAge, and (3) examine β2M expression and activated pathways in response to PA at the single-cell level in mice. This approach bridges observational and molecular research, offering insights into the mechanisms through which PA influences aging and identifying β2M as a potential therapeutic target for promoting healthy aging.

## Results

### Characteristics of participants

**Fig. 2:**
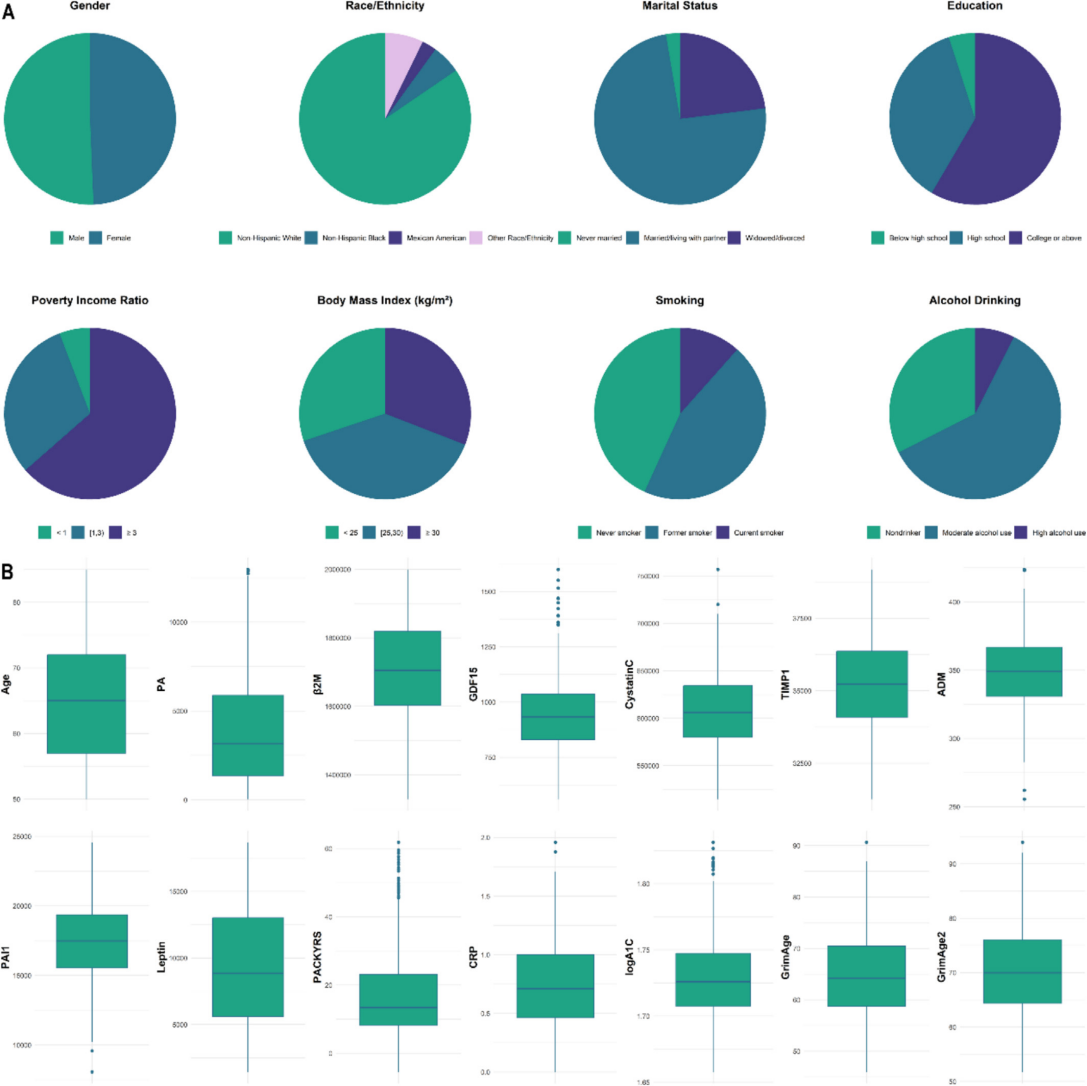
Characteristics of study participants and distribution of DNA-predicted biomarkers. Notes: (A) Pie charts illustrating the distribution of categorical variables, including gender, race/ethnicity, marital status, education, poverty income ratio, BMI, smoking status, and alcohol consumption. (B) Boxplots showing the distribution of continuous variables, including age, PA, β2M, and DNAm-predicted biomarkers such as GDF15, Cystatin C, TIMP1, ADM, Leptin, PACKYRS, CRP, logA1C, GrimAge, and GrimAge2.

A total of 936 participants were included in the study. Accounting for the weighted sampling methodology, this represented a weighted population of 3,782,115. Fig. 2 provides an overview of the characteristics of the participants included in the primary analysis. The sample was nearly evenly distributed by gender, with 50.64 % of participants identifying as male and 49.36 % as female. The majority of participants (84.54 %) were Non-Hispanic White, followed by smaller proportions of Non-Hispanic Black (5.48 %), Mexican American (2.74 %), and other racial/ethnic groups (7.23 %). Most participants were married or living with a partner (74.33 %), while 23.01 % were widowed or divorced, and only 2.65 % had never been married. Regarding education, 58.44 % of participants had achieved a college-level education or higher, with 36.58 % having completed high school, and 4.98 % falling below a high school level. The majority (63.54 %) had a poverty income ratio greater than 3, indicating higher income levels, while 30.67 % fell into the moderate income category, and 5.80 % had a ratio below 1, suggesting lower income. For body mass index (BMI), the distribution was relatively even across categories, with 30.16 % of participants having a BMI below 25, 38.94 % in the overweight range (BMI 25–30), and 30.90 % classified as obese (BMI ≥ 30). Smoking status showed that former smokers accounted for 45.23 % of the sample, followed by never smokers (43.19 %) and current smokers (11.58 %). In terms of alcohol consumption, the majority were moderate drinkers (60.06 %), while 32.49 % were nondrinkers, and 7.45 % engaged in high alcohol use. Detailed distribution of category variable is shown in Fig. 2**A**. For continuous variables (Fig. 2**B**), the average age of participants was 62.54 years. Physical activity levels, measured in METs per week, averaged 7352.52. Several DNAm-predicted biomarkers were also measured. For instance, the mean value for β2M was 1,682,136.33, while the growth differentiation factor 15 (GDF15) averaged 911.43. Additionally, the predicted cystatin C (CystatinC) was 598,043.12, and the predicted adrenomedullin (ADM) was 346.05. Index like GrimAge and GrimAge2 showed means of 62.76 years and 68.25 years, respectively, suggesting that the participants’ DNAm profiles reflected these predicted ages. Detailed characteristics of participants in this study can be found in Table S1.

### Association between physical activity and DNAm-predicted PhenoAge

Table 1 represents the association between PA and DNAm-predicted PhenoAge, with results presented as β and 95 % CI to show the percentage change in Log-based DNAm-predicted PhenoAge per 100 MET-minutes increase in PA per week. Among all participants, the crude model showed a β of −0.015 (95 % CI: −0.025, −0.005, p = 0.005), while Model 1, adjusted for gender and race, showed a β of −0.016 (95 % CI: −0.026, −0.006, p = 0.004), and Model 2, adjusted for multiple covariates, yielded a β of −0.014 (95 % CI: −0.026, −0.001, p = 0.034). Gender-stratified analysis indicated significant negative associations in males across all models, with Model 2 showing a β of −0.016 (95 % CI: −0.028, −0.003, p = 0.017), while in females, significant associations were found only in the crude and Model 1, but not in Model 2 (β = -0.014, 95 % CI: −0.032, 0.003, p = 0.095). BMI-stratified analysis showed no significant associations in the BMI < 25 kg/m^2^ group, while those with a BMI of 25–30 kg/m^2^ showed a β of −0.017 (95 % CI: −0.031, −0.002, p = 0.031) in Model 1, and those with a BMI ≥ 30 kg/m^2^ had a β of −0.023 (95 % CI: −0.045, −0.001, p = 0.045) in Model 2. These results suggest that PA is significantly associated with a reduction in DNAm-predicted PhenoAge, particularly among males and individuals with higher BMI.

**Table 1:** Associations between physical activity and Log-based DNAm-prediced PhenoAge.

	Crude model^a^		Model 1^b^		Model 2^c^	
	β (95 % CI)	*p-value*	β (95 % CI)	*p-value*	β (95 % CI)	*p-value*
Total participants	−0.015(−0.025,-0.005)	0.005	−0.016(−0.026,-0.006)	0.004	−0.014(−0.026,-0.001)	0.034

*Gender*						
Male	−0.013(−0.023,-0.004)	0.007	−0.014(−0.024,-0.005)	0.006	−0.016(−0.028,-0.003)	0.017
Female	−0.019(−0.035,-0.003)	0.022	−0.019(−0.035,-0.003)	0.023	−0.014(−0.032, 0.003)	0.095

*BMI (kg/m^2^)*						
<25	−0.011(−0.030,0.008)	0.126	−0.011(−0.032,0.009)	0.256	−0.010(−0.029, 0.009)	0.257
[25, 30)	−0.015(−0.031,0.000)	0.057	−0.017(−0.031,-0.002)	0.031	−0.016(−0.037, 0.005)	0.122
≥30	−0.018(−0.037,0.001)	0.057	−0.018(−0.038,0.001)	0.068	−0.023(−0.045,-0.001)	0.045

Notes: ^a^Crude model, no covariate was adjusted. ^b^Model 1, gender and race were adjusted. ^c^Model 2, gender, race, marital status, education, poverty status, body mass index, smoking status, and alcohol use status were adjusted. CI, confidence interval.

**Fig. 3:**
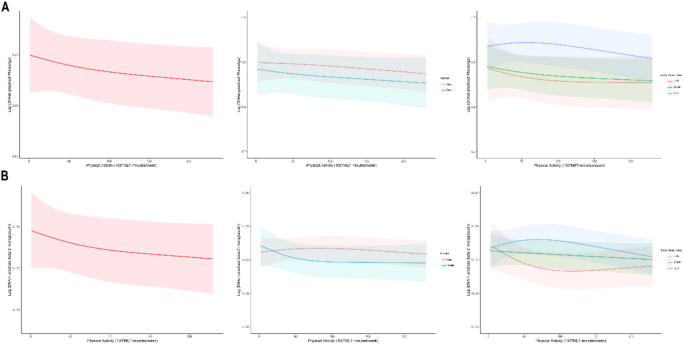
Dose-response relationship between physical activity and DNAm-predicted PhenoAge (A) and β2M (B). Notes: (A) RCS plots showing the relationship between physical activity (MET-minutes/week) and Log DNAm-predicted PhenoAge across all participants (left), stratified by gender (middle), and stratified by BMI categories (right). The red, blue, and green lines represent different groups, with shaded areas indicating 95 % confidence intervals. (B) RCS plots depicting the relationship between physical activity (MET-minutes/week) and Log DNAm-predicted β2M levels. Plots are presented for all participants (left), stratified by gender (middle), and stratified by BMI categories (right). Shaded areas denote 95 % confidence intervals.

Fig. 3**A** demonstrates a negative association between PA and DNAm-predicted PhenoAge by restricted cubic spline (RCS) analyses, with higher levels of PA linked to lower PhenoAge. The middle subplot shows that this negative association is consistent across genders, but the decline is slightly steeper for males (blue line) compared to females (red line), suggesting that PA might have a stronger impact on reducing PhenoAge in males. In the right subplot, stratification by BMI reveals that individuals with a BMI ≥ 30 (blue line) experience the steepest decline in PhenoAge with increasing PA, while those with a BMI < 25 (red line) exhibit a less pronounced effect.

### Association between physical activity and DNAm-predicted biomarkers

Table 2 presents the associations between PA and Log-based β2M. In the total sample, a significant negative association was observed, with a β of −0.006 (95 % CI: −0.012 to −0.001, p = 0.032) in the fully adjusted model, indicating that increased PA is associated with lower β2M levels. Gender-stratified analysis showed a stronger association in females (β = -0.007, 95 % CI: −0.015 to 0.000, p = 0.039) than in males (β = -0.006, 95 % CI: −0.013 to 0.001, p = 0.071). Among BMI categories, the strongest association was found in participants with a BMI ≥ 30 (β = -0.013, 95 % CI: −0.027 to 0.000, p = 0.049). These results suggest that the beneficial effects of PA on β2M may vary by gender and BMI.

**Table 2:** Associations between physical activity and Log-based β2M levels.

	Crude model^a^		Model 1^b^		Model 2^c^	
	β (95 % CI)	*p-value*	β (95 % CI)	*p-value*	β (95 % CI)	*p-value*
Total participants	−0.008(−0.013,-0.002)	0.010	−0.007(−0.013,-0.002)	0.014	−0.006(−0.012,-0.001)	0.032

*Gender*						
Male	−0.006(−0.013,0.000)	0.057	−0.006(−0.013, 0.000)	0.045	−0.006(−0.013, 0.001)	0.071
Female	−0.009(−0.016,-0.002)	0.020	−0.009(−0.016,-0.002)	0.014	−0.007(−0.015,0.000)	0.039

*BMI (kg/m^2^)*						
<25	−0.003(−0.014,0.008)	0.587	−0.003(−0.014,0.008)	0.592	−0.002(−0.013, 0.010)	0.756
[25, 30)	−0.009(−0.019,0.001)	0.088	−0.008(−0.018,0.002)	0.121	−0.007(−0.017, 0.003)	0.136
≥30	−0.012(−0.022,-0.001)	0.021	−0.011(−0.021,0.000)	0.047	−0.013(−0.027, 0.000)	0.049

Notes: ^a^Crude model, no covariate was adjusted. ^b^Model 1, gender and race were adjusted. ^c^Model 2, gender, race, marital status, education, poverty status, body mass index, smoking status, and alcohol use status were adjusted. CI, confidence interval. The results show the percentage change in the β2M per 100 MET-minutes/week increase in physical activity, expressed as % change (95 % CI: lower bound, upper bound).

Fig. 3**B** illustrates the relationship between physical activity (PA) and Log DNAm-predicted β2M levels using RCS. The overall trend (left subplot) shows a negative association, with higher PA levels linked to lowerβ2M levels. By gender (middle subplot), both males and females exhibit a decrease in DNAm-predicted β2M with increased PA, but the decline is sharper in females (red line) compared to males (blue line). Stratification by BMI (right subplot) reveals that individuals with a BMI ≥ 30 (blue line) experience the steepest decline in DNAm-predicted β2M with increasing PA, while those with a BMI < 25 (red line) show a weaker association.

This study also examined the association between PA and other DNAm-derived epigenetic biomarkers (Supplementary Table 2). PA showed a significant inverse association with Log (Cystatin C), indicating a 0.004 decrease (β = 0.004, 95 % CI: −0.008 to 0.000, p = 0.030) in Cystatin C per 100 MET-minutes/week increase in PA. Other biomarkers exhibited non-significant trends, such as Log (TIMP1) (β = −0.003, 95 % CI: −0.006 to 0.001, p = 0.093), which showed a borderline negative association, while Log (Leptin) approached significance with a positive association (β = 0.022, 95 % CI: −0.004 to 0.048, p = 0.089). Additionally, both Log (GrimAge) (β = −0.007, 95 % CI: −0.016 to 0.002, p = 0.125) and Log (GrimAge2) (β = −0.006, 95 % CI: −0.014 to 0.001, p = 0.096) demonstrated marginally negative but non-significant associations with PA. Several biomarkers, including Log (GDF15) (β = 0.001, p = 0.846), Log (PACKYRS) (β = -0.038, p = 0.355), and Log (CRP) (β = −0.020, p = 0.330), did not show significant associations, indicating that the increase in PA was not associated with these biomarkers.

### Mediation analysis of the association between physical activity, DNAm-predicted β2M, and PhenoAge

**Fig. 4:**
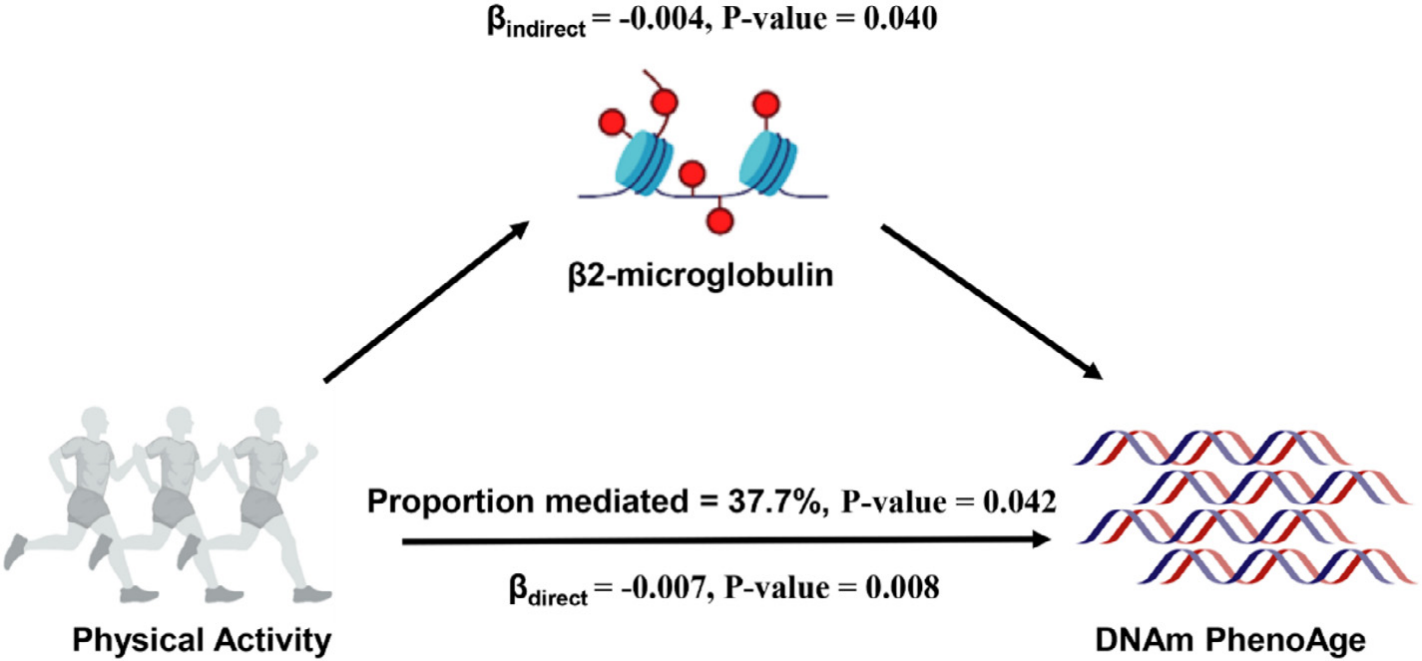
Path diagram of the mediation analysis between physical activity, β2M, and PhenoAge. Notes: The arrows represent causal pathways, highlighting the partial mediating role of β2M in the PA-PhenoAge relationship.

The mediation analysis (Fig. 4) evaluated whether the effect of PA on PhenoAge was mediated by β2M. The total effect of PA on PhenoAge was significant (β = −0.011, 95 % CI: −0.020 to −0.005, p = 0.002), suggesting that higher PA levels are associated with younger PhenoAge. A significant mediation effect was observed (β = −0.004, 95 % CI: −0.009 to −0.001, p = 0.040), indicating that β2M partially mediated the relationship between PA and PhenoAge. The direct effect remained significant (β = −0.007, 95 % CI: −0.014 to −0.002, p = 0.008), while the proportion of the effect mediated by β2M was 37.67 % (p = 0.042). These findings suggest that β2M plays a substantial role in linking PA with reduced biological aging, while the direct effect of PA on PhenoAge remains robust.

### Exercise-induced immune modulation and β2M expression in aging: insights from molecular and cellular pathways analysis

**Fig. 5:**
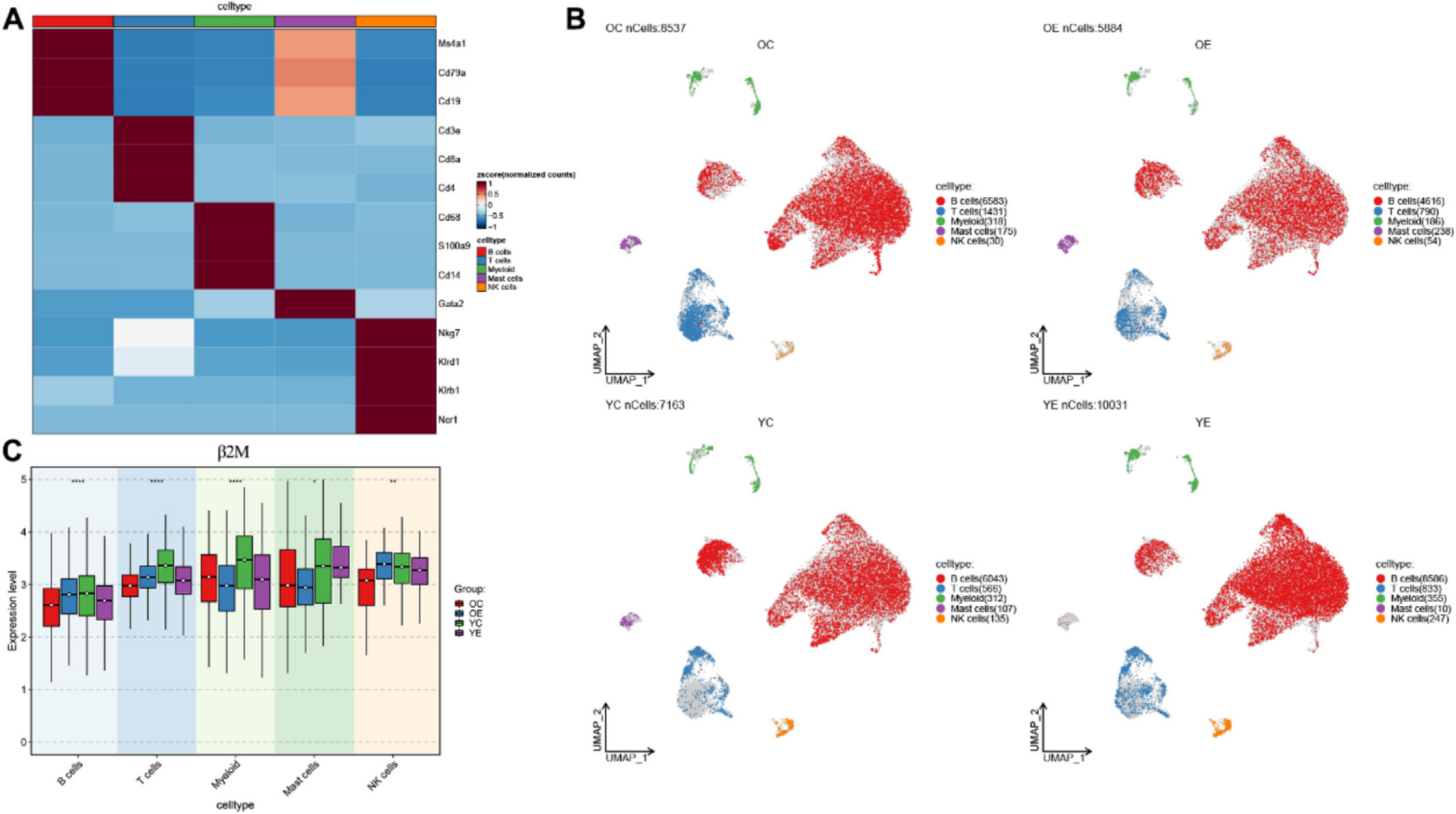
Marker gene expression, immune cell distribution, and β2M transcriptional dynamics across experimental groups. (A) Heatmap showing z-score-normalized expression levels of marker genes across five immune cell types (B cells, T cells, myeloid cells, mast cells, NK cells). Each marker gene is selectively expressed in specific cell types, highlighting their cellular identity. (B) UMAP visualization of single-cell RNA-seq data from four experimental groups: OC, OE, YC, and YE. Colors represent cell types, and YE exhibits an enriched population of B cells and other immune cells compared to OC. (C) Boxplots illustrating β2M expression levels across cell types and experimental groups. β2M expression is highest in B cells and myeloid cells, with exercise (YE and OE) enhancing expression compared to aging (OC).

In peripheral blood immune cells from young and aged mice subjected to exercise or control conditions, cellular subpopulations were further annotated in subsequent analyses. The heatmap highlights the expression levels of characteristic marker genes across immune cell types, including B cells (Ms4a1, Cd79a, Cd19), T cells (Cd3e, Cd4, Cd8a), myeloid cells (Cd68, S100a9, Cd14), mast cells (Gata2), and NK cells (Nkg7, Klrd1, Klrb1, Ncr1) (Fig. 5**A**). The UMAP plot depicts the distribution and composition of blood immune cells in the four experimental groups of mice: Old Control (OC), Old Exercise (OE), Young Control (YC), and Young Exercise (YE). These immune cell populations include B cells, T cells, myeloid cells, mast cells, and NK cells (Fig. 5**B**). Overall, B cells dominated across all groups, followed by T cells and myeloid cells, while mast cells and NK cells were present in lower numbers with distinct distribution patterns. The YE group exhibited the highest total cell count, with a significant increase in B cells, whereas the T cell and NK cell counts were notably lower in the old groups (OC and OE) compared to the young groups. Significant changes in cell composition were observed in the exercise groups (OE and YE), suggesting potential anti-inflammatory and immune regulatory effects of exercise. The boxplot revealed the expression characteristics of the β2M gene across different immune cell types (Fig. 5**C**), showing that β2M expression was significantly elevated in the YE group, particularly in B cells and myeloid cells, indicating that exercise might influence immune function by regulating β2M expression.

**Fig. 6:**
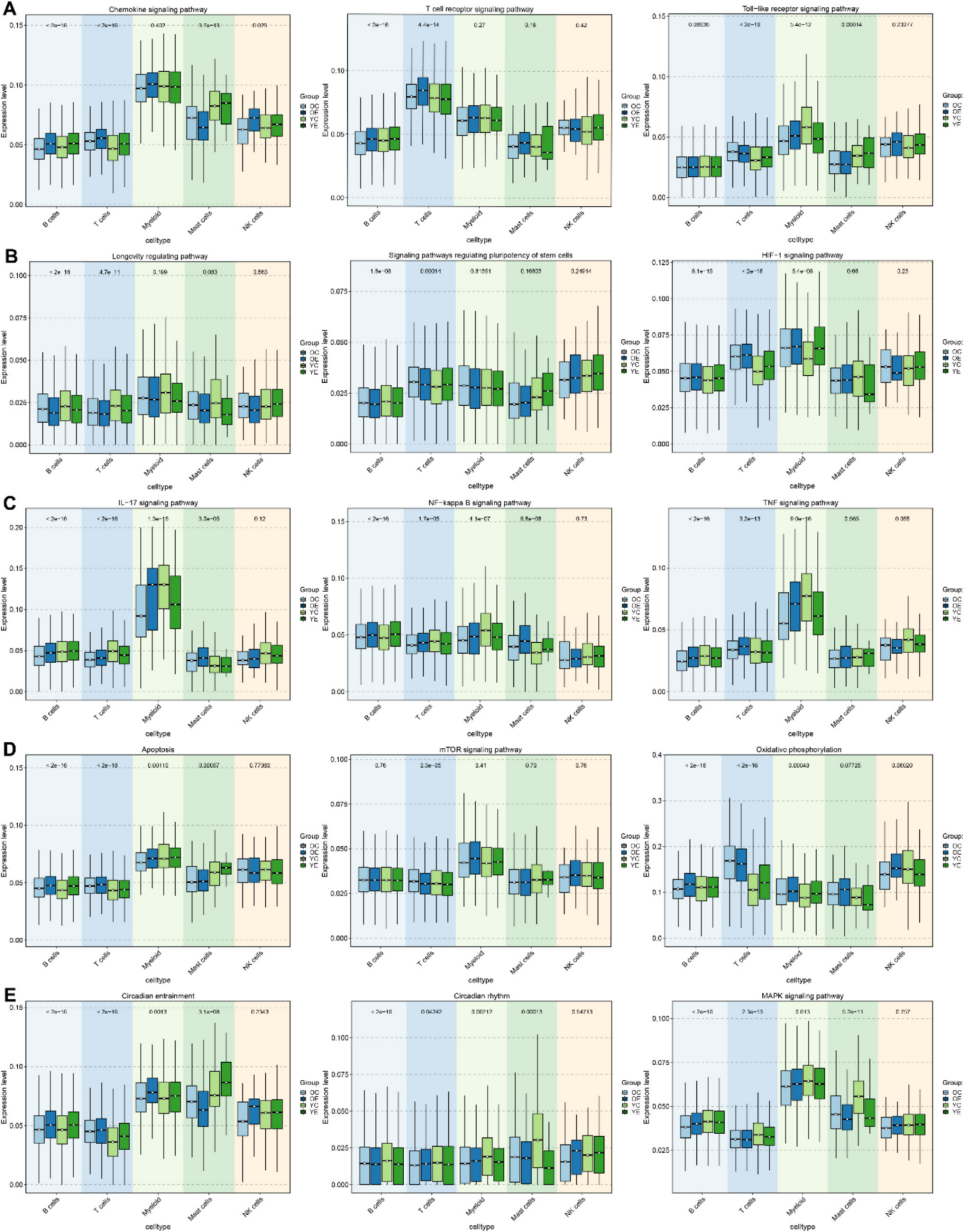
Expression levels of pathway activities across experimental groups and cell types. Notes: Boxplots represent median expression levels with interquartile ranges for each pathway, stratified by cell type and experimental group. Significance levels are annotated above the plots. (A) Boxplots showing the expression levels of immune signaling pathways, including chemokine signaling, T cell receptor signaling, and Toll-like receptor signaling, across experimental groups (OC, OE, YC, YE) and cell types. (B) Boxplots displaying the activity of longevity regulation and stem cell pluripotency pathways across experimental groups and cell types. (C) Boxplots representing the expression levels of inflammatory signaling pathways, including IL-17 signaling, NF-κB signaling, and TNF signaling, across experimental groups and cell types. (D) Boxplots showing metabolic and mitochondrial pathway activities, such as mTOR signaling and oxidative phosphorylation, across experimental groups and cell types. (E) Boxplots illustrating the expression levels of circadian rhythm-related pathways, including circadian entrainment and MAPK signaling, across experimental groups and cell types.

The boxplots in Fig. 6 further illustrate the expression levels of key signaling pathways across different immune cell types (B cells, T cells, myeloid cells, NK cells, and mast cells) and experimental groups (OC, OE, YC, YE), focusing on immune signaling, longevity regulation, inflammatory response, mitochondrial function, and circadian rhythm. Overall, the OC group exhibited the lowest expression levels across most pathways, reflecting characteristics of immune senescence and metabolic decline, while the YE group showed the highest expression levels, indicating significant exercise-induced enhancements. Immune signaling pathways, including chemokine signaling, T cell receptor signaling, and Toll-like receptor signaling, showed reduced activity in the OC group but were significantly elevated in the exercise groups, particularly in myeloid cells and B cells (Fig. 6**A**). Longevity-related pathways, such as those regulating stem cell pluripotency, displayed notable upregulation in the YE group across multiple cell types, highlighting exercise’s potential to promote cellular rejuvenation (Fig. 6**B**). Inflammatory pathways, including IL-17 and NF-κB signaling, were generally lower in the OC group and demonstrated increased activity in the exercise groups, although the extent of change varied by cell type (Fig. 6**C**). Mitochondrial and metabolic pathways, such as oxidative phosphorylation, mTOR signaling, and apoptosis, exhibited the most substantial activity in the YE group, with pronounced increases in myeloid cells and B cells (Fig. 6**D**). Circadian rhythm pathways also showed reduced activity in the OC group and significantly higher activity in the YE group, particularly in B cells and myeloid cells (Fig. 6**E**).

## Discussions

Combined with human and mice studies, this study highlights the significant relationship between PA and reduced DNAm-predicted PhenoAge, with β2M serving as a partial mediator. Notably, the associations were stronger in males and individuals with higher BMI, suggesting that PA may exert more pronounced anti-aging effects in these subgroups, potentially due to their higher baseline levels of inflammation and metabolic dysregulation. Our findings not only confirm the anti-aging effects of PA but also provide mechanistic insights into how PA modulates biological aging by profoundly influencing pathways related to immunity, inflammation, aging, mitochondrial function, and circadian rhythm.

Firstly, our analysis demonstrated that higher PA levels were associated with a significant reduction in DNAm-predicted PhenoAge, consistent with previous research showing the anti-aging benefits of regular PA [[34], [35], [36]]. The dose–response analysis revealed that the decline in PhenoAge with increased PA was more pronounced in males and individuals with a higher BMI. These findings suggest that PA may be particularly effective in reducing biological aging in overweighed populations, potentially due to their higher levels of systemic inflammation [37,38] or metabolic dysregulation [39,40]. This is consistent with evidence that PA improves obesity and reduces systemic inflammation and improves insulin sensitivity, both of which contribute to slower biological aging [41,42].

Secondly, in our dose–response analysis, we observed a non-linear association between weekly PA volume and DNAm PhenoAge. This “threshold-then-plateau” pattern suggests that relatively modest increases in PA—particularly among previously sedentary adults—produce disproportionate epigenetic benefits, whereas additional activity above guideline levels yields diminishing returns. Comparable dose–response shapes have been reported for all-cause mortality and cardiometabolic outcomes, reinforcing the public-health importance of moving people “from none to some” rather than exclusively promoting very high training volumes [[43], [44], [45]]. Biologically, early-phase gains may arise from rapid improvements in insulin sensitivity, adipose tissue browning, and suppression of the renal–inflammatory β_2_-microglobulin axis. By contrast, higher exercise doses elicit adaptive hormetic responses—transient oxidative stress, autophagy, and inflammatory rebound [46,47].

Moreover, β2M was identified as a key mediator linking PA and PhenoAge. β2M is elevated in states of chronic inflammation and is implicated in immune senescence [21,48]. Our study showed that increased PA was significantly associated with lower β2M levels, and mediation analysis revealed that reductions in β2M explained 37.67 % of the association between PA and PhenoAge. These results align with findings that PA mitigates inflammation by reducing pro-inflammatory cytokines and improving immune function [49,50]. Importantly, the direct effect of PA on PhenoAge remained significant even after accounting for β2M, suggesting additional pathways through which PA exerts anti-aging effects, such as epigenetic regulation or mitochondrial function [51,52]. Beyond β2M, only DNAm-predicted cystatin-C showed a modest inverse association with PA, whereas TIMP-1, leptin, CRP, and GDF-15 were null (Supplementary Table 2). This pattern suggests that PA may preferentially modulate renal-inflammatory axes (β2M, cystatin-C) rather than broad senescence-secretory proteins. Null results could also reflect lower assay precision of certain CpG clocks or insufficient between-person variation in a relatively healthy sample.

Additionally, single-cell RNA-sequencing analyses in mice provided mechanistic evidence for the cellular and molecular pathways involved in the anti-aging effects of PA. Exercise significantly increased the abundance of B cells and improved the activity of immune and metabolic pathways, including chemokine signaling and oxidative phosphorylation. Importantly, the exercise groups, particularly the young exercise group, exhibited reduced expression of inflammatory pathways such as IL-17 and NF-κB signaling, which are known contributors to immune senescence and chronic inflammation [53,54]. PA was also shown to restore circadian rhythm pathways disrupted by aging, as evidenced by increased pathway activity in the YE group. Circadian rhythm disruption is a hallmark of aging, contributing to immune dysfunction and metabolic dysregulation [[55], [56], [57]]. By restoring circadian rhythm, PA may help maintain homeostasis in key physiological systems, further supporting its role in promoting healthy aging [58,59]. These findings underscore the systemic benefits of regular PA, extending beyond individual biomarkers to include broader physiological processes.

Our findings also indicated an approximately 50 % reduction in total myeloid-cell abundance in the OE group compared with the OC group (Fig. 5B). Myeloid populations—especially pro-inflammatory macrophages and antigen-presenting dendritic cells—are known amplifiers of “inflamm-ageing” and have been implicated in frailty, sarcopenia and cardio-metabolic decline [60]. Lifelong or late-life endurance training can attenuate this trajectory by reducing the pool of senescence-associated, CCR2^+^/Ly6C^hi^ inflammatory monocytes and by shifting tissue macrophages toward an M2-like, tissue-repair phenotype [61]. Exercise has also been reported to dampen NF-κB signalling in dendritic cells, thereby lowering systemic IL-6 and TNF-α levels in aged hosts [62]. Although our single-cell dataset did not have sufficient depth to resolve myeloid subsets (e.g., classical vs. non-classical monocytes, M1 vs. M2 macrophages), the global contraction of the myeloid compartment we observe is consistent with these earlier reports and may partly explain the reduced β2-microglobulin levels seen in physically active participants.

This study has several notable strengths. First, it is based on a large population sample, providing strong epidemiological evidence for the relationship between PA and DNAm-predicted biological aging. Second, it employs the novel use of epigenetic biomarkers, such as DNAm-predicted PhenoAge and β2M, offering advanced insights into the biological underpinnings of aging. Third, β2M is identified as a mediator linking PA and biological aging, with its role validated through complementary findings from both human population data and animal models, enhancing the mechanistic understanding of the PA-aging relationship. As for clinical implications, β2M could be a simple lab test to follow lifestyle-related shifts in biological ageing alongside PA counselling. We recommend longitudinal trials that change the amount of exercise and regularly measure both DNAm-β2M and PhenoAge; studies combining exercise programs with drug treatments aimed at β2M would also be valuable.

Despite its strengths, this study has limitations. The cross-sectional nature of the human data restricts causal inference between PA, β2M, and DNAm-predicted aging markers. Additionally, potential residual confounding factors, such as dietary habits or genetic predispositions, could influence the observed associations. Furthermore, because the NHANES 1999–2002 cycle lacks a full panel of soluble senescence-associated secretory phenotype (SASP) proteins, we could not assemble a multidimensional ageing index that integrates epigenetic clocks with circulating SASP markers. In addition, because our measurements are confined to circulating β2-microglobulin, we cannot determine how changes in peripheral β2M translate to corresponding alterations within the brain; the mechanistic linkage between the periphery and the central nervous system therefore remains unresolved. Finally, the pathway interpretations derived from our single-cell RNA-seq data rely solely on transcript abundance; we did not validate key proteins or their post-translational modifications with techniques such as western blotting, so the functional signal-transduction network remains to be confirmed.

## Conclusion

This study demonstrates that PA is associated with reduced DNAm-predicted PhenoAge, with β2M identified as a partial mediator. Stronger associations in males and individuals with higher BMI highlight the potential for targeted interventions in populations with elevated baseline inflammation or metabolic dysfunction. Insights from scRNA-seq analyses in mice revealed that PA modulates immune, inflammatory, mitochondrial, and circadian pathways, emphasizing the systemic benefits of PA in counteracting biological aging. These findings support PA as a non-pharmacological strategy for healthy aging and establish β2M as an important link in the PA-aging relationship. Future research should focus on population-specific effects of PA to optimize interventions and further explore β2M-targeted molecular and pharmacological strategies to enhance anti-aging outcomes.

## Methods

### Study participants

Our participants were derived from the National Health and Nutrition Examination Survey (NHANES) population. NHANES is a large-scale study conducted by the National Center for Health Statistics (NCHS), part of the Centers for Disease Control and Prevention, aimed at assessing the overall health and dietary habits of the U.S. population not living in institutional settings. This survey uses a complex, stratified, multistage probability sampling approach to ensure a representative sample. Data are gathered through in-home interviews, assessments at mobile examination centers, and laboratory analyses. The survey protocol is approved by the NCHS Ethics Review Board, and all participants sign written informed consent forms prior to participating. For this particular study, we focused on DNAm predicted biomarkers’ data from the 1999–2002 NHANES cycles (NHANES published this data on July 31, 2024, and DNAm data is only availale in this year cycle) [63]. The sample included individuals aged 50 and above who had provided blood samples for DNA analysis. Initially, 4449 participants were included, with a random subset of non-Hispanic White individuals and all eligible non-Hispanic Black, Mexican American, other Hispanic, and other racial groups. After excluding participants missing DNAm data, physical activity records, or other covariates, the final sample for analysis consisted of 936 participants.

### Physical activity assessment

Participants’ PA was measured using self-reported data on their involvement in various activities, such as walking, swimming, and playing sports, over the past month. Data were collected through the Physical Activity Questionnaire (PAQ), which gathered detailed information on daily habits, leisure activities, and sedentary behaviors [64]. Participants provided information on the number of days they participated in specific activities over the past 30 days, as well as the typical duration of each session in minutes. NHANES classified activities as moderate or vigorous, assigning each a metabolic equivalent of task (MET) score based on its intensity [65,66]. These MET values, derived from a recognized compendium of physical activities, were linked to 62 activities listed on the PAQ section. The total PA level (reflected by MET-min/week) for each individual was calculated by summing the minutes of reported activity and multiplying by the corresponding MET value for each activity [67,68]. Ultimately, this cumulative value can be divided by 100 to quantify the final PA volume as 100 MET-min/week.

### DNAm-predicted biomarkers assessment

In this study, the extraction of key DNA methylation-based biomarkers, including GrimAge, GrimAge2, adrenomedullin (ADM), β2M, and others, follows a detailed methodology involving multiple stages of data processing and analysis [69]. These biomarkers, which we are particularly interested in, provide insights into biological aging and mortality predictions. GrimAge and GrimAge2, both based on Horvath's DNA methylation models [70,71], predict mortality in whole blood, with GrimAge2 being an updated version of the original GrimAge mortality predictor. Other biomarkers, such as ADM and β2M, predict the time of death using methylation levels, respectively, within the framework of GrimAge and GrimAge2. Similarly, cystatin C (CystatinC) and growth differentiation factor 15 (GDF15) use methylation data to estimate time of death for the same predictive models.

The preprocessing of these signals involved several steps [72]. Raw IDAT files were first converted into methylated and unmethylated signals, followed by background correction and normalization across the two-color fluorescent channels used in the array. Any samples that displayed extreme outlier characteristics were excluded, ensuring the integrity of the remaining dataset. Color correction helped to adjust for technical variation, and normalization was performed using beta-mixture quantile normalization (BMIQ) to account for probe design bias.

In addition to these biomarkers, we focused on several others: leptin (Leptin), which uses leptin methylation to predict mortality; log-transformed hemoglobin A1c (logA1C) and high-sensitivity C-reactive protein (CRP), which predict time of death using their respective methylation levels, specifically for GrimAge2. Pack years of smoking (PACKYRS) predicts smoking exposure, while plasminogen activator inhibitor 1 (PAI1) and tissue inhibitor of metalloproteinases 1 (TIMP1) use their methylation data to estimate mortality risk. These methylation values were applied to generate predictions regarding an individual's mortality and aging processes.

Throughout the analysis, missing methylation values were imputed using reference datasets, and BMIQ was employed to correct any biases [63]. The final biomarker values, such as GrimAge, ADM, and β2M, were then calculated by applying predefined coefficients to the methylation data from the corresponding CpG sites.

### DNAm-predicted PhenoAge assessment

Following the extraction of these key biomarkers, PhenoAge was another critical DNA methylation-based indicator that we assessed to provide a broader understanding of biological aging. PhenoAge, derived from a model developed by Levine et al., is designed to estimate an individual's phenotypic age [7], which more accurately reflects their biological aging compared to chronological age. Referring to previous creators' methods [7,8], biomarkers were extracted using a similar process to the other indicators: methylation data from whole blood samples was first preprocessed by correcting for background noise, normalizing the signals, and removing outliers to maintain data integrity.

PhenoAge calculation relied on predefined coefficients linked to specific CpG sites associated with aging and various physiological markers. After preprocessing, the methylation levels at these CpG sites were applied to the PhenoAge model, producing a score that estimates biological age. This score, which integrates the effects of aging across different biological systems, provides a measure of aging and mortality risk. Like other biomarkers, PhenoAge values were adjusted through BMIQ to account for probe bias, and any missing data were imputed using reference datasets. This method ensured that PhenoAge, along with other biomarkers, offered a detailed and reliable assessment of an individual’s biological aging trajectory.

### Statistical analysis of NHANES population

The statistical analyses followed the protocols outlined by the CDC, as detailed on their website (https://www.cdc.gov/nchs/nhanes/tutorials/default.aspx). Continuous data were expressed as weighted means and standard errors (Mean ± SE), while categorical data were represented as weighted percentages. To test for significance, the survey-weighted Chi-square test was used for categorical variables, and survey-weighted linear regression was applied for continuous variables.

In this study, covariates are background characteristics and lifestyle factors that might influence both PA and biological aging, and therefore need to be adjusted for to avoid confounding. We incorporated several important covariates, including demographic factors such as gender, race/ethnicity, educational attainment, and marital status. Other covariates included poverty-to-income ratio, body mass index, smoking habits, and alcohol consumption levels.

To assess the link between PA and DNA methylation-based biomarkers and PhenoAge, three regression models were developed. The crude model, did not adjust for any covariates. The Model 1 controlled for gender and race, while the third model adjusted for a broader set of variables, including gender, race, marital status, education, poverty level, BMI, smoking status, and alcohol intake. A fully adjusted multivariable linear regression (Model 2) was used to further explore the associations between PA and DNA methylation-predicted biomarkers. The 95 % confidence intervals (CI) for β were calculated from the standard errors of the regression coefficients, which in turn were derived using the Taylor series linearization method accounting for NHANES’s complex sampling design. The p-values reported are the significance levels from the regression, testing the null hypothesis that the true β equals zero.

Restricted cubic splines with 3-knots were used to assess potential non-linear relationships between PA and the predicted outcomes, providing a flexible method to explore dose–response effects [73]. In addition, mediation analysis was performed to examine whether the effect of PA on PhenoAge was mediated through other DNAm-based β2M. We fitted survey-weighted regression models for both the mediator and the outcome, then quantified the indirect (mediated) effect and direct effect of PA using non-parametric bootstrapping with 5 000 resamples; percentile bootstrap 95 % confidence intervals and two-sided p-values were derived from the empirical distribution of the indirect effect. Additionally, subgroup analyses (e.g., stratified by sex and BMI) and interaction tests were carried out to explore these relationships across different subgroups.

The analyses were performed using R software (version 4.3.3), and statistical significance was determined by a two-tailed p-value of less than 0.05.

### Molecular and cellular pathways analysis

In this study, C57BL/6J male mice were categorized into four experimental groups: Young Control (YC), Young Exercise (YE), Old Control (OC), and Old Exercise (OE), comprising 2-month-old and 16-month-old mice, respectively. The exercise intervention involved a voluntary running model [74], where mice in the exercise groups were housed in cages equipped with wireless-monitored running wheels for 12 months, allowing unrestricted access. Sedentary control mice were kept in standard cages without running wheels. At the conclusion of the experiment, the mice underwent systemic perfusion with physiological saline via cardiac infusion, and peripheral blood was collected from 5 mice per group for subsequent analysis.

Single-cell RNA sequencing (scRNA-seq) was employed to assess the effects of exercise on peripheral blood cells. Cell suspensions were processed using the 10x Genomics Chromium platform, encapsulating single cells into droplet emulsions with the Chromium Single-Cell instrument. The sequencing data, obtained from a previously published dataset [75], were subjected to stringent quality control, retaining 31,615 cells for downstream analyses (Supplementary Fig. 1A–D). Quality metrics such as the number of detected genes per cell (nFeature_RNA), total RNA count per cell (nCount_RNA), and mitochondrial RNA percentage (percent.mt) were assessed across experimental groups. Violin plots (Supplementary Fig. 1A, B) reveal that the YE group exhibited the highest transcriptional diversity, while the OC group demonstrated reduced diversity, indicating potential age-related transcriptional decline. Highly variable genes critical for downstream clustering, such as Hbb-bs and S100a8, were identified (Supplementary Fig. 1**C**). The uniform manifold approximation and projection (UMAP) analysis (Supplementary Fig. 1D) revealed transcriptionally unique cell populations, with greater immune diversity and activity observed in the YE and YC groups compared to the OC group, highlighting the systemic effects of exercise on transcriptional profiles. The “Harmony” R package was utilized for integrating and normalizing data across samples.

To explore the molecular pathways and biological processes influenced by exercise, Gene Ontology (GO) (https://geneontology.org/) and Kyoto Encyclopedia of Genes and Genomes (KEGG) (https://www.genome.jp/kegg) enrichment analyses were conducted, focusing on results with p adjust < 0.05. Pathway activity levels were quantified using the “Singscore” R package. Data visualization included heatmaps and gene expression clustering generated with the pheatmap R package, while additional plots were created with ggplot2. Statistical comparisons between groups were performed using two-tailed t-tests and Wilcoxon tests.

## Ethics approval and consent to participate

The National Health and Nutrition Examination Survey (NHANES) is a publicly accessible database approved by the Institutional Review Board (IRB) of the National Center for Health Statistics. All participants provided written informed consent during their participation in the national survey. As secondary analysis does not require additional IRB approval, this study was exempt from further ethical review and approval.

## Declaration of competing interest

The authors declare that they have no known competing financial interests or personal relationships that could have appeared to influence the work reported in this paper.
