# Supplementary material for: Exploring the role of β2-microglobulin in the relationship between physical activity and DNAm-predicted PhenoAge: Evidence from a population-based and mice single-cell RNA-sequencing study
Source: J Adv Res. 2025 Nov 22;86:743–55. doi: 10.1016/j.jare.2025.11.047 (PMC13453862; doi:10.1016/j.jare.2025.11.047)
Supplement: Supplementary file 1 [file mmc4.pdf]

National Health and Nutrition Examination Survey 1999-2000 and 2001-2002 DNA Methylation Array and Epigenetic Biomarkers Data Documentation

First Published: July 31, 2024 Last Revised: N/A

Component Description

DNA methylation (DNAm) is one type of potentially reversible epigenetic modification, characterized by the addition of a methyl group to a cytosine DNA base without changing the DNA sequence.[1] DNAm is prevalent in gene promoter regions and may influence gene expression. In certain cases, DNAm is also mitotically heritable, allowing for the maintenance of cell-specific gene expression throughout an organism's lifetime.[2] An important use of DNAm data is in the production of epigenetic biomarkers,[3] which are associated with various health outcomes and mortality.[4–8]

DNAm was measured from biospecimens collected from NHANES participants during the 1999-2000 and 2001-2002 cycles. DNAm was measured using Illumina EPIC BeadChip arrays. Methylation data matrices were produced, pre-processed, and normalized. DNAm derived epigenetic biomarkers predicting chronological age, phenotypic age, telomere length, pace of aging, mortality, and mitotic cell turnover rate were produced.

This documentation contains information for the NHANES DNAm array derived data for 1999-2000 and 2001-2002. It describes the samples, laboratory methods, and bioinformatics procedures used to produce the final DNAm data files.

Eligible Samples

A selection of adults aged 50 years and over surveyed in 1999-2000 or 2001-2002 who had blood collected for DNA purification were eligible. The sample includes a random selection of approximately one half of eligible non-Hispanic White participants and all eligible non-Hispanic Black, Mexican American, other Hispanic, and other race participants.

Description of Laboratory Methodology

DNA was extracted from whole blood, and specimens were stored at -80°C. The DNAm assay was performed in the laboratory of Dr. Yongmei Liu at Duke University. Bisulfite conversion of DNA was carried out using manufacturer's recommendations. 500ng of DNA was bisulfite treated using a Zymo EZ DNA Methylation kit (cat# D5001, Zymo Research, Irvine, CA, USA) using PCR conditions for Illumina's Infinium Methylation assay (95°C for 30 seconds, 50°C for 60 minutes x16 cycles). Data were produced on the Illumina Infinium MethylationEPIC BeadChip v1.0 (cat# WG317-1001, Illumina, San Diego, CA, USA). A total of 4 µL of bisulfite converted DNA was hybridized to the Illumina BeadChip using manufacturer protocols. The samples were denatured and amplified overnight for 20-24 hours. Fragmentation, precipitation, and resuspension of the samples followed overnight incubation, prior to hybridization to the EPIC BeadChip for 16-24 hours. The BeadChip was then washed to remove any unhybridized DNA and labelled with nucleotides to extend the primers to the DNA. Following the Infinium HD Methylation protocol, the BeadChip was imaged using the Illumina iScan system (Illumina, San Diego, CA, USA).

Bioinformatics Procedures

The following section briefly describes the pre-processing and normalization steps to

produce the DNAm epigenetic biomarkers from the Illumina EPIC BeadChip array IDAT files. All pre-processing and DNAm epigenetic biomarker production were completed in the RStudio environment[9] [version 2023.03.01 build 446] using the R language[10] [version 4.3.1]. Specific code used to produce these data files can be found in **Appendix 1 & Appendix 2**.

1. **IDAT file pre-processing into methylated and unmethylated files**

The output of the Illumina EPIC BeadChip arrays were IDAT files, which are images of the chip scans in a green and red fluorescent channel. DMAP files provided by Illumina denoted the genomic location of the specific signals accompanying the IDATs. For initial pre-processing, these files were used to transform the red and green intensity signals into the methylated and unmethylated signals.

2. **Color correction and background subtraction**

The Illumina EPIC BeadChip arrays contained a set of control probes designed to determine system background levels, probe specificity, and bisulfite conversion, staining, extension, hybridization, and target removal efficiency. These probes were used to calculate and then subtract background levels. Additionally, to account for intensity variation between the two-color channels used, color correction was performed by normalizing across color channels.

3. **Epigenetic biomarker production**
   a. **Sample outlier detection and removal**
   Outlier samples were removed if the median intensity values of both the methylated and unmethylated channels were <10.5.
   b. **Imputation**
   We assessed the percentage of probes within each epigenetic biomarker that was missing in more than 5% of samples. Imputation was done in two different ways according to the DNAm epigenetic biomarker creators. For the Horvath[11], Hannum[12], SkinBlood[13], GrimAge[14], GrimAge2[15], and PhenoAge[16] biomarkers, a gold standard reference data set produced by Horvath was used where the mean DNAm beta values were imputed for missing data values of each DNAm site. For the telomere[17], Yang[18], Zhang[19], Lin[20], Weidner[21], VidalBralo[22], and DunedinPoAM[23] biomarkers, the NHANES dataset was used to calculate mean CpG measures to impute missing values.
   c. **Normalization**
   The Illumina EPIC BeadChip array utilized a two-probe design tailored to measure methylation in low and high density CpG regions resulting in a probe type bias, which was normalized using the beta mixture quantile (BMIQ) method[24] which fit a 3-state beta mixture model, transformed the state-membership of type 2 probes to fit quantiles of type 1, and transformed hemi-methylated probes conformally. Original BMIQ methods[24] were used for most epigenetic biomarkers. For the Horvath[17], Hannum[12], SkinBlood[13], GrimAge[14], GrimAge2[15], and PhenoAge[16] biomarkers, a modified BMIQ version was used where instead of normalizing type 2 probes against type 1 probes, it was done against a gold standard produced by Horvath as per the epigenetic biomarkers' creators' methods.
   d. **DNAm epigenetic biomarker predictions**
   The coefficients provided by the authors of each epigenetic biomarker (Horvath[17], Hannum[12], SkinBlood[13], PhenoAge[16], telomere[17], Yang[18], Zhang[19], Lin[20], Weidner[21], VidalBralo[22], DunedinPoAM[23], GrimAge[14], and GrimAge2[15]) were applied to the corresponding subset of probes to produce a score for each participant.

e. **Cell type proportion prediction**
As NHANES samples were sourced from blood, a heterogenous tissue, DNAm was used to predict cell type proportion for pre-processing and analysis purposes using a regression calibration algorithm[25]. Specifically, the IDOL probe subset was used in combination with the FlowSorted.Blood.EPIC_ref reference dataset on the NHANES data to predict cell type proportions using the "estimateCellCounts2" function from the *immunomethylomics/FlowSorted.Blood.EPIC* package[26–28]. A proportional estimate of six cell types (neutrophils, monocytes, B-lymphocytes, natural killer cells, $CD4^+$, and $CD8^+$ T-cells) was produced for each participant.

f. **Sample mismatch identification and removal**
Three analyses were performed to identify any sample mismatches: 1) samples with more than two standard deviations of discrepancy between chronological age and DNAm predicted age, 2) samples with more than two standard deviations of discrepancy between lab measured cell type proportion and DNAm predicted cell type proportion measures, and 3) samples with unexpected XY chromosomal ploidy based on mean differences of raw DNAm measures. Samples mismatched based on all three criteria were removed.

4. **EWAS beta matrix production**

a. **Normalization**
Functional normalization[29] was applied to the beta matrix. This method utilized the control probes present on the array designed to account for technical variation encountered with these data.

b. **Removing outlier samples**
Outlier samples were determined by utilizing several different methods and then removing those detected by more than one method. The methods were:

i. **Lumi[30]:** Outliers were determined when the measures within a sample's distance to the center was larger than two median distances to the center of all other samples.

ii. **WateRmelon pcout[31]:** Samples with significant weight, distance, and scatter from all samples, as determined by robust principal components methods, were determined as outliers.

iii. **locFDR[12]:** Samples with an FDR<0.2 standardized based on the squared distance of the first PC from the population mean using principal component analysis were labelled as outliers. This process was done iteratively until no further outliers were detected.

c. **Removing outlier probes**
Probes with a performance p-value $>1x10^{-16}$ were considered outliers and were removed[32].

d. **Probe filtering**
Control probes that do not have a strong signal above background and bind multiple sites within the genome were removed prior to analysis:

i. **SNP probes:** 59 single nucleotide polymorphism (SNP) probes designed to uniquely identify an individual on the EPIC array were used to confirm technical replicates from the same samples.

ii. **Poorly performing probes:** Probes which could not be measured in ≥ 1% of samples were removed.

iii. **XY chromosome probes:** Due to the uneven distribution of X and Y chromosomes among the population, probes designed to measure sites located in

these chromosomes were normalized and preprocessed separately.

iv. **Polymorphic probes:** Probes containing SNPs either at the CpG site being measured or at the site of the single base pair extension which has a minor allele frequency (MAF) greater than or equal to 1% were removed. Additionally, DNAm site measurements predicted to be impacted by proximal SNPs by Pidsley et. al.,[31] and Price et. al.,[33] were also removed.

v. **Cross-hybridizing probes:** Probes predicted in silico to bind elsewhere in the genome than intended were identified as cross-hybridizing probes to aid in future analysis[33,34].

e. **Technical variation correction**
ComBat[35] was applied to remove residual variation attributed to technical sources of variation including plate, chip, and row number using both parametric and non-parametric methods.

Analytic Notes

The DNAm data files can be linked to the other NHANES 1999-2000 and 2001-2002 data files using the participant identifier (SEQN). The data dictionary for the DNAm files can be found in Table 1.

Special sample weights are required to analyze these data properly. Specific sample weights for this subsample, WTDN4YR, are included in these data files and should be used when analyzing these data. The sample weights created for this file used the examination sample weight, i.e., WTMEC4YR, as the base weight. The base weight was adjusted for additional nonresponse to these array tests and re-poststratified to the population total using sex, age, and race/Hispanic origin. Participants who were part of the eligible population but who did not provide a blood specimen for DNA, did not have sufficient volume of DNA specimens, or did not give consent for their specimens to be used for future research are included in the file, but they have a sample weight assigned "0" in their records. For additional information on weighted analyses refer to the NHANES analytic guidelines.

Flow Chart – Sample to Data Files

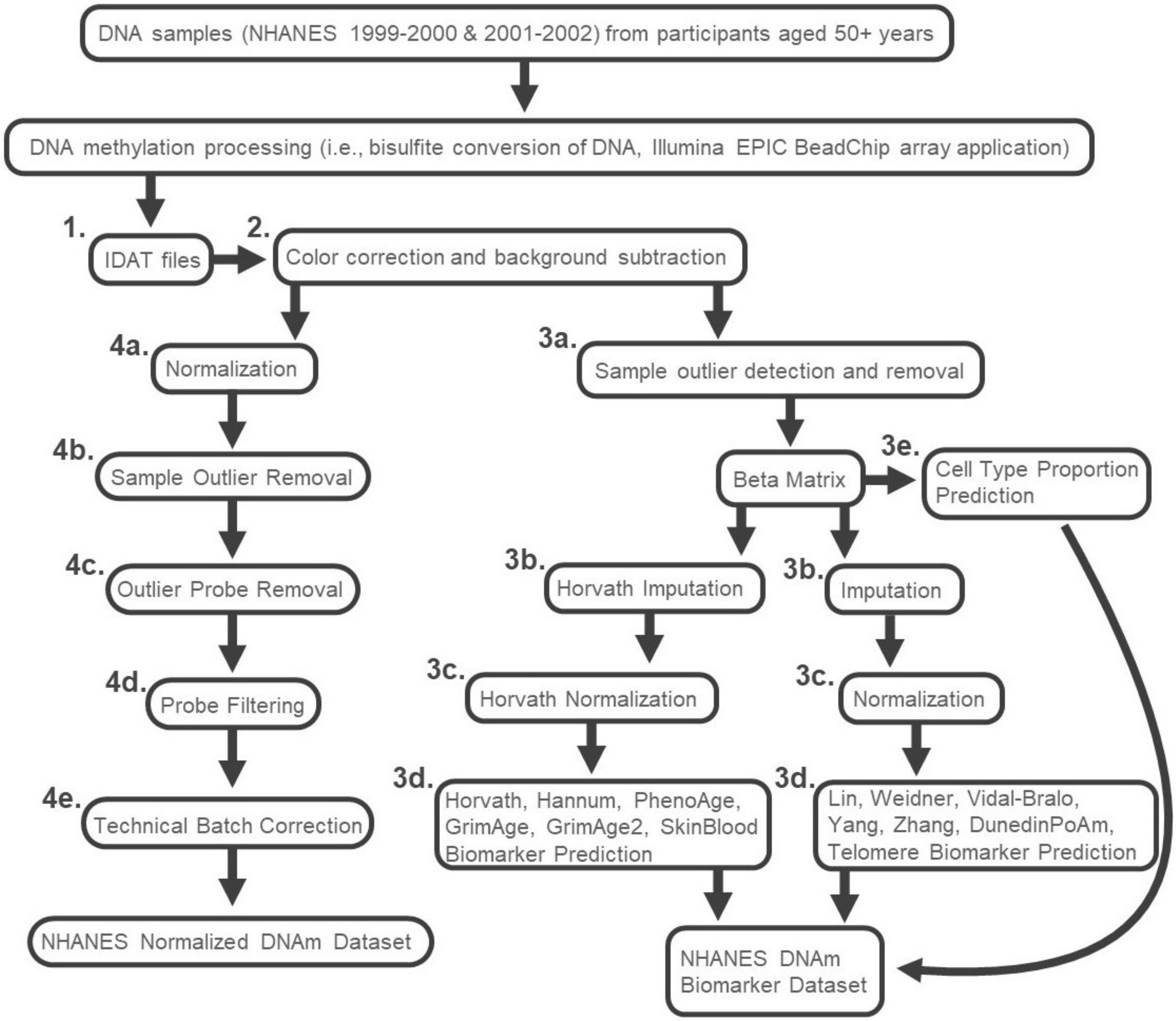
DNA samples (NHANES 1999-2000 & 2001-2002) from participants aged 50+ years
DNA methylation processing (i.e., bisulfite conversion of DNA, Illumina EPIC BeadChip array application)
1.
IDAT files
2.
Color correction and background subtraction
4a.
Normalization
4b.
Sample Outlier Removal
4c.
Outlier Probe Removal
4d.
Probe Filtering
4e.
Technical Batch Correction
NHANES Normalized DNAm Dataset
3a.
Sample outlier detection and removal
Beta Matrix
3e.
Cell Type Proportion Prediction
3b.
Horvath Imputation
3b.
Imputation
3c.
Horvath Normalization
3c.
Normalization
3d.
Horvath, Hannum, PhenoAge, GrimAge, GrimAge2, SkinBlood Biomarker Prediction
3d.
Lin, Weidner, Vidal-Bralo, Yang, Zhang, DunedinPoAm, Telomere Biomarker Prediction
NHANES DNAm Biomarker Dataset

Data Files

File Name	Description	Data File	Mode of Access
**DNAm Epigenetic Biomarker**	DNAm derived epigenetic biomarkers predicting chronological age, phenotypic age, telomere length, pace of aging, mortality, and mitotic cell turnover rate	dnmepi.sas7bdat	Public
**Normalized and Pre-processed DNAm**	Normalized beta values where the rows are CpG sites and the columns are sample names with values ranging from 0 to 1.	DNAm-CpG-Sites.csv	Restricted

Variable List

Table 1. DNAm Epigenetic biomarker variable list

Variable	Label
**HorvathAge**[11]	Horvath DNA methylation predicted chronological age in 51 tissues.
**HannumAge**[12]	Hannum DNA methylation predicted chronological age in whole blood.
**SkinBloodAge**[13]	Horvath DNA methylation predicted chronological age in skin and blood derived tissues.
**PhenoAge**[16]	Levine DNA methylation predicted phenotypic age in whole blood.
**LinAge**[20]	Lin DNA methylation predicted chronological age in whole blood.
**WeidnerAge**[21]	Weidner DNA methylation predicted chronological age in whole blood.
**VidalBraloAge**[22]	Vidal-Bralo DNA methylation predicted chronological age in whole blood.
**YangCell**[18]	Yang DNA methylation predicted mitotic cell division in whole blood.
**ZhangAge**[19]	Zhang DNA methylation predicted chronological age in whole blood and saliva.
**GrimAgeMort**[14]	Horvath DNA methylation predicted mortality in whole blood.
**GrimAge2Mort**[15]	Horvath updated DNA methylation predicted mortality in whole blood.
**DunedinPoAm**[23]	Belsky DNA methylation predicted pace of aging in whole blood.
**HorvathTelo**[17]	Horvath DNA methylation predicted telomere length in leukocytes.
**ADMMort**[14]	DNA methylation predicted adrenomedullin used to predict time of death for GrimAge and GrimAge2.
**B2MMort**[14]	DNA methylation predicted beta-2 microglobulin used to predict time of death for GrimAge and GrimAge2.
**CystatinCMort**[14]	DNA methylation predicted cystatin C used to predict time of death for GrimAge and GrimAge2.

**GDF15Mort**[14]	DNA methylation predicted Beta-2 growth differentiation factor 15 used to predict time of death for GrimAge and GrimAge2.
**LeptinMort**[14]	DNA methylation predicted leptin used to predict time of death for GrimAge and GrimAge2.
**logA1CMort**[15]	DNA methylation predicted hemoglobin A1c used to predict time of death for GrimAge2.
**CRPMort**[15]	DNA methylation predicted high sensitivity C-reactive protein used to predict time of death for GrimAge2.
**PACKYRSMort**[14]	DNA methylation predicted pack years of smoking used to predict time of death for GrimAge and GrimAge2.
**PAI1Mort**[14]	DNA methylation predicted plasminogen activation inhibitor used to predict time of death for GrimAge and GrimAge2.
**TIMP1Mort**[14]	DNA methylation predicted tissue inhibitor metalloproteinase 1 used to predict time of death for GrimAge and GrimAge2.
**XY_Estimation**	DNA methylation predicted sex chromosomes (1 = XX, 2 = XY).
**CD8TPP**[26–28]	DNA methylation predicted CD8+ T-cell proportion in blood sample.
**CD4TPP**[26–28]	DNA methylation predicted CD4+ T-cell proportion in blood sample.
**NKcell**[26–28]	DNA methylation predicted natural killer cell proportion in blood sample.
**Bcell**[26–28]	DNA methylation predicted B-cell proportion in blood sample.
**MonoPP**[26–28]	DNA methylation predicted monocyte proportion in blood sample.
**NeuPP**[26–28]	DNA methylation predicted neutrophil proportion in blood sample.

Appendix 1: Code for NHANES 1999-2000 and 2001-2002 DNA methylation array pre-processing and normalization

1 Libraries

```
library("minfi")
library("BiocGenerics")
library("Biobase")
library("scales")
library("reshape2")
library("crayon")
library("withr")
library("ggplot2")
library("matrixStats")
library("FDb.InfiniumMethylation.hg19")
library("foreach")
library("iterators")
library("locfit")
library("bumphunter")
library("methylumi")
library("gplots")
library("limma")
library("marray")
library("lumi")
library("wateRmelon")
library("plyr")
library("sva")
library("IlluminaHumanMethylationEPICanno.ilm10b2.hg19")
library("IlluminaHumanMethylationEPICanno.ilm10b4.hg19")
library("IlluminaHumanMethylationEPICmanifest")
library("RPMM")
library("WGCNA")
library("impute")
library("FlowSorted.Blood.EPIC") library("reticulate")
library("Metrics")
library("vroom")
library("dbplyr")
library("locfdr")
library("magrittr")
library("dendextend")
library("quantro")
```

2 Creating MethyLumiSet Object

Code for reading in iDats to create a MethyLumiSet object.

```
path <- "~/NHANES_idats"
sampleInfo <- read.csv("~/NHANES_Samplesheet.csv", header = T, row.names = 1, skip = 7)
sampleInfo$Basename <- paste(sampleInfo$Sentrix_ID, sampleInfo$Sentrix_Position, sep="_")
sampleInfo$Chip_Position <- sampleInfo$Basename
sampleInfo$Basename <- file.path(path, sampleInfo$Basename)
NHANES_MS <- readEPIC(barcodes = sampleInfo$Chip_Position, pdat = sampleInfo, n = T, oob = T,
idatPath = path)
sampleNames(NHANES_MS) <- sampleInfo$Sample_Group
save(NHANES_MS, file = "~/NHANES_MS.RData")
```

3 Quality Control Check

Code for analyzing the control probes to see how the array performed and to see if there are any outlier samples present.

```
load("~/NHANES_qcraw.RData") # This was produced from NHANES_DNAm_Biomarker_Code
outliers <- NHANES_qcraw$Sample_ID[NHANES_qcraw$Threshold <= 10.5]
```

4 Normalization - Funnorm

Function implementing functional normalization pre-processing for Illumina methylation microarrays[34].

4.1 FunNorm PCA

Determining how many principal components (PC) to include in the Funnorm function, which is defined as the number of PCs which accounts for at least 90% of the variance:

```
load("~/NHANES_RG_Raw.RData") # This was produced from NHANES_DNAm_Biomarker_Code
source("~/PCA_forFunNorm.R")
controlPCA <- PCA_forFunNorm(NHANES_RG_Raw)
ctrlPCASum <- summary(controlPCA)$importance
colnames(ctrlPCASum)[ctrlPCASum[3,] >= 0.9][[1]] #
```

4.2 FunNorm Application

Applying the normalization method and replacing the beta matrix into the methylumi set object:

```
NHANES_RG_FN <- preprocessFunnorm(NHANES_RG_Raw, nPCs = 3)
save(NHANES_RG_FN, file = "~/NHANES_RG_FN.RData")
NHANES_MS <- NHANES_MS[featureNames(NHANES_MS) %in% rownames(getBeta(NHANES_RG_FN))]
NHANES_RG_FN <- NHANES_RG_FN[featureNames(NHANES_RG_FN) %in% rownames(betas(NHANES_MS))]
NHANES_RG_betas <- getBeta(NHANES_RG_FN)
NHANES_MS_betas <- betas(NHANES_MS)
NHANES_RG_betas <- NHANES_RG_betas[order(match(rownames(NHANES_RG_betas),
rownames(NHANES_MS_betas))),]
betas(NHANES_MS) <- NHANES_RG_betas
save(NHANES_MS, file = "~/NHANES_MS.RData")
```

5 Adding EPIC Platform Annotation

Adding additional EPIC annotation.

```
load("~/EPIC_Annotation_Complete.RData") probe_names <-
rownames(NHANES_MS)
EPIC_Ann <- EPIC_Annotation_Complete[EPIC_Annotation_Complete$Name %in% probe_names,]
probe_names_2 <- rownames(EPIC_Ann)
NHANES_MS <- NHANES_MS[featureNames(NHANES_MS) %in% probe_names_2,]
EPIC_Ann <- EPIC_Ann[match(rownames(NHANES_MS), rownames(EPIC_Ann)),]
fData(NHANES_MS) <- EPIC_Ann
save(NHANES_MS, file = "NHANES_MS.RData")
```

6 Detecting Outlier Samples

6.1 Detect Outlier in Lumi

The outlier detection was based on the distance from the sample to the center (average of all samples). The assumption of the outlier detection is that there is only one single cluster and the distance from the sample to the center is Gaussian distributed[30]. An outlier is detected when its distance to the center is larger than a certain threshold. The threshold is calculated as Th * median

distances to the center.

```
NHANES_detout <- detectOutlier(betas(NHANES_MS))
save(NHANES_detout, file = "~/NHANES_detout.RData")
```

6.2 Outlier Detection Pcout - wateRmelon

This is a wateRmelon package[32] outlier detection method wrapped in the function "pcout". Based on robustly sphered data, semi-robust principal components were computed, which are needed for determining distances for each observation. Separate weights for location and scatter outliers were computed based on these distances. The combined weights were used for outlier identification.

```
pcout_NHANES <- outlyx(NHANES_MS)
pcout_NHANES$Sample_ID <- rownames(pcout_NHANES)
pcout_NHANES[which(pcout_NHANES$outliers == T),]
```

6.3 locFDR Outlier Detector

Code for outlier detection by[12]. We used PC analysis to identify and remove outlier samples based on the squared distance of its first PC from the population mean. The z-statistic was converted to a false-discovery rate using the Benjamini-Hochberg procedure[36]. Samples with FDR <0.2 were designated as outliers and removed. This was performed iteratively until no outliers remained.

```
meta <- pNHANES(NHANES_MS)
PCA<-as.data.frame(unclass(princomp(betas(NHANES_MS))$loadings))
zstat_comp1<-(PCA$Comp.1-mean(PCA$Comp.1))/sd(PCA$Comp.1)
w <- locfdr(as.matrix(zstat_comp1)) fdr <-
as.data.frame(w$fdr) rownames(fdr) <-
rownames(PCA) outmeta<-meta[which(fdr
<= 0.1),] outmeta$PCA<-"FDR<=0.1"
inmeta<-meta[which(!(fdr <= 0.1)),]
inmeta$PCA<-"FDR>=0.1"
outliers<-as.character(outmeta$Sample_Group)
meta_out<-rbind(outmeta,inmeta)
meta_out <- meta_out[order(match(colnames(betas(NHANES_MS)), meta_out$Sample_Group)),]
```

6.4 pfilter - WateRmelon

Code for detecting samples having ≥ 5% of sites with a detection p-value greater than $1x10^{-16}$ and labeling them as outliers.

```
NHANES.pf<-pfilter(NHANES_MS, pnthresh = 1e-16, perc = 5)
```

6.5 Removing Outlier Samples

```
NHANES_MSR <- NHANES_MS[, sampleNames(NHANES_MS) %in% Outlier]
save(NHANES_MSR, file = "~/NHANES_MSR.RData")
```

7 Probe Filtering

Probe filtering prior to analysis included removing control probes, probes which do not have a strong signal above background, and probes which bind multiple sites within the genome.

7.1 Investigating SNP Probes

There are 59 SNP probes utilized to observe sample mismatches and to ensure replicates cluster together.

```
NHANES_MS.rs <- as.data.frame(getSnpBeta(NHANES_RG_Raw))
meta <- pData(NHANES_MS)
CB_Dist <- dist(t(as.matrix(NHANES_MS.rs))) CB_hc
<- hclust(CB_Dist, 'ave')
```

```
CB_hc_cluster<-data.frame(cluster=cutree(CB_hc,k =6),
                          states=factor(CB_hc$labels,levels=CB_hc$labels[CB_hc$order]))
CB_hc_cluster <- join(CB_hc_cluster, meta, by = "Sample_Group")
CBB_Dend <- as.dendrogram(CB_hc)
CBB_Dend %>% set("labels_cex", 0.2) %>% plot(main = "SNP Dendrogram")
```

7.2 Removing Poorly Performing Probes

For values with 3 or more reads the average measurement was taken and a p-value was provided for each value. If the detection p-value was above $1x10^{-16}$ in 1% of samples or more, that probe was also removed.

```
source("~/BadProbe_Function.R")
NHANES_RG_Raw_B <- NHANES_RG_Raw[,sampleNames(NHANES_RG_Raw) %in%
                          sampleNames(NHANES_MSR)]
Bad_Probe_List <- Bad_Probes(NHANES_RG_Raw_B, samp_percentage = 1)
nbadDet <- unlist(Bad_Probe_List$nbadDetP)
nbadAvgbeta <-        unlist(Bad_Probe_List$nbadAvgbeta)
NHANES_MSR <- NHANES_MSR[!featureNames(NHANES_MSR) %in% c(nbadAvgbeta, nbadDet)]
save(NHANES_MSR, file = "~/NHANES_MSR.RData")
```

7.3 Filtering Polymorphic and Cross-Hybridizing Probes

7.3.1 Polymorphic Probes

Probes were removed which contained a SNP either at the CpG site being measured or at the site of the single base pair extension which has a minor allele frequency greater than or equal to 1%.

```
fdat <- fData(NHANES_MSR)
CpG <- rownames(fdat)[which(fdat$CpG_maf >= 0.01)]
SBE <- rownames(fdat)[which(fdat$SBE_maf >= 0.01)]
NHANES_MSR <- NHANES_MSR[!featureNames(NHANES_MSR) %in% c(CpG, SBE)]
```

7.3.2 Cross-Hybridizing Probes

XY probes predicted to cross-hybridize to autosomes were removed. Pulling XY probes predicted to cross-hybridize to autosomes:

```
load("~/EPIC_Annotation_Complete.RData")
X_bind_Aut <- rownames(EPIC_Annotation_Complete)
[EPIC_Annotation_Complete$CH_450_Aut == "Yes" & EPIC_Annotation_Complete$CHR == "X"]
Y_bind_Aut <- rownames(EPIC_Annotation_Complete)
[EPIC_Annotation_Complete$CH_450_Aut == "Yes" & EPIC_Annotation_Complete$CHR == "Y"]
NHANES_MSR.xy<- NHANES_MSR.xy[!featureNames(NHANES_MSR.xy) %in% c(X_bind_Aut, Y_bind_Aut)]
save(NHANES_MSR.xy, file = "~/NHANES_MSR.xy.RData")
```

Pulling autosomal probes predicted to cross-hybridize to XY chromosome:

```
Aut_XY_Binders <- rownames(EPIC_Annotation_Complete)
[EPIC_Annotation_Complete$CH_450_XY == "Yes"]
NHANES_MSR<- NHANES_MSR[!featureNames(NHANES_MSR)%in%Aut_XY_Binders]
save(NHANES_MSR, file = "~/NHANES_MSR.RData")
```

8 Technical Variation Correction

Code to determine if there is any association between patterns of DNAm and technical batch.

```
table(pData(NHANES_MSR)$Sample_Plate) # X plates
pData(NHANES_MSR)$Sentrix_Row <- substr(pData(NHANES_MSR)$Sentrix_Position,1,3)
table(pData(NHANES_MSR)$Sentrix_Position) # X rows. unique(pData(NHANES_MSR)$Sentrix_ID)
table(pData(NHANES_MSR)$Sentrix_ID) # X chips
```

8.1 PCA Investigation

Code to run PCA to determine whether major variability in the data is related to technical batches.

```
meta <- pData(NHANES_MSR)
meta_categorical <- meta[, c(2,5,7,9)] meta_continuous
<- meta[, c(11,15)]
colnames(meta_categorical) <- c("Plate", "Chip", "Row", "Sex")
colnames(meta_continuous) <- c("BMI", "Age")
ord <- c(seq(1:sum(ncol(meta_categorical), ncol(meta_continuous))))
source("~/PCA_Plot_Function.R") PCA_full<-prcomp(betas(NHANES_MSR))
PCA_Plot(PCA_full, nPCs = 30, type = "All", label.y_size = 18, label.x_size = 12)
```

8.2 Plate Correction

ComBat[35] corrects for batch effects in microarrays for studies with smaller batch sizes (<25).

```
NHANESNC1 <- NHANES_MSR
NHANESNC1_M <- exprs(NHANESNC1)
mval.combat1 <- ComBat(NHANESNC1_M, NHANESNC1$Sample_Plate)
betas(NHANESNC1) <- m2beta(mval.combat1)
save(NHANESNC1, file = "~/NHANESNC1.RData")
```

8.3 Chip Row Correction

Repeating for removing variation attributed to the row on the chip the sample was placed.

```
NHANESNC2 <- NHANESNC1 NHANESNC2_M
<- exprs(NHANESNC2)
mval.combat2<- ComBat(NHANESNC2_M, NHANESNC2$Sentrix_Row)
betas(NHANESNC2)<- m2beta(mval.combat2)
save(NHANESNC2, file = "~/NHANESNC2.RData")
```

8.4 Chip Correction

Re-running based on which chip the samples were run.

```
NHANESNC3 <- NHANESNC2 NHANESNC3_M
<- exprs(NHANESNC3)
mval.combat3<- ComBat(NHANESNC3_M, NHANESNC3$Sentrix_ID)
betas(NHANESNC3)<- m2beta(mval.combat3)
save(NHANESNC3, file = "~/NHANESNC3.RData")
```

9 Replicate Removal

Removing replicate samples.

```
rep_out <- c("samp1_rep1")
NHANESNC <- NHANESNC[, !sampleNames(NHANESNC)%in%rep_out]
save(NHANESNC, file = "~/NHANESNC.RData")
```

Appendix 2: Code for NHANES 1999-2000 and 2001-2002 DNA

methylation array biomarker production

1 Libraries

```
library("minfi")
library("BiocGenerics")
library("Biobase")
library("scales")
library("reshape2")
library("crayon")
library("withr")
library("ggplot2")
library("matrixStats")
library("FDb.InfiniumMethylation.hg19")
library("foreach")
library("iterators")
library("locfit")
library("bumphunter")
library("methylumi")
library("gplots")
library("limma")
library("marray")
library("lumi")
library("wateRmelon")
library("plyr")
library("IlluminaHumanMethylationEPICanno.ilm10b2.hg19")
library("IlluminaHumanMethylationEPICanno.ilm10b4.hg19")
library("IlluminaHumanMethylationEPICmanifest")
library("RPMM")
library("WGCNA")
library("impute")
library("FlowSorted.Blood.EPIC")
library("reticulate")
library("Metrics")
library("DunedinPoAm45")
library("DunedinPoAm38")
library("vroom")
```

2 Functions & Future Use Objects

2.1 Cell Type Reference

```
hub <- ExperimentHub()
query(hub, "FlowSorted.Blood.EPIC")
FlowSorted.Blood.EPIC_ref <- hub[["EH1136"]]
save(FlowSorted.Blood.EPIC_ref, file ="~/FlowSorted.Blood.EPIC_ref.RData")
```

2.2 DNAm Epigenetics Biomarker Coefficients

Code for making a single table containing all epigenetic biomarker coefficients and probe names required for prediction.

```
Horvath_coef <- read.csv("Horvath_coef.csv")
      horvathCoef <- Horvath_coef[,2]
      names(horvathCoef) <- Horvath_coef[,1]
hannum_coef_dat <- read.csv("datCoefHannum.csv")
```

```
        hannumCoef <- hannum_coef_dat[,2]
        names(hannumCoef) <- hannum_coef_dat[,1]
skinbloodCoef_dat <- read.csv("datSkinClock.csv")
        skinbloodCoef <- skinbloodCoef_dat[,2]
        names(skinbloodCoef) <- skinbloodCoef_dat[,1]
PhenoAgeCoef_dat <- read.csv("datPhenoAge.csv")
        phenoageCoef <- PhenoAgeCoef_dat[,2]
        names(phenoageCoef) <- PhenoAgeCoef_dat[,1]
load("coefTL.rda")
        telomereCoef <- coefTL[,2]
        names(telomereCoef)  <-  coefTL[,1]
load("Yang_CpGID.rdata")
        yangCoef <- epiTOCcpgs.v
load("zhangCoef.RData")
        zhangCoef <- as.data.frame(zhangCoef)
        ZhangCoef <- zhangCoef[,2]
        names(ZhangCoef) <- zhangCoef[,1]
lin_Coef <- read.csv("linCoef_dat.csv")
        linCoef <- lin_Coef[,2]
        names(linCoef) <- lin_Coef[,1]
# The following values were obtained from Lin et al. 2016
weidnerCoef <- c(111.83, -64.57, -42.57, 75.15)
        names(weidnerCoef) <- c("(intercept)", "cg02228185", "cg25809905", "cg17861230")
vidalbralo_Coef <- read.csv("VidalBraloCoefs.csv")
        vidalbraloCoef <- vidalbralo_Coef[,2]
        names(vidalbraloCoef) <- vidalbralo_Coef[,1]
Dunedin38Probes <- unlist(DunedinPoAm38::getRequiredProbes()$DunedinPoAm_38)
save( horvathCoef, hannumCoef, skinbloodCoef, phenoageCoef, telomereCoef, yangCoef, ZhangCoef,
linCoef, weidnerCoef, vidalbraloCoef, Dunedin38Probes, file = "DNAmAgeCoef.RData")
```

2.3 DNAm_Biomarker_Probe_Count_NG Function:

Function to calculate the number of probes missing from all DNAm epigenetic biomarkers except GrimAge, GrimAge 2, and their components (the code for these is proprietary).

```
DNAm_Biomarker_Probe_Count_NG   <-   function(betas){
        biomarker <- c('horvath', 'hannum','skinblood', 'phenoage', 'telomere', 'yang','zhang',
        'DunedinPoAm38', 'lin', 'weidner', 'vidalbralo')
        probes_in_biomarker  <- c(length(names(horvathCoef))-1,  length(names(hannumCoef)),
                length(names(skinbloodCoef)), length(names(phenoageCoef))-1,
                length(names(telomereCoef))-1, length(yangCoef), length(names(ZhangCoef))-1,
                length(DunedinPoAm38::getRequiredProbes()$DunedinPoAm_38), length(names(linCoef))-1,
                length(names(weidnerCoef))-1, length(names(vidalbraloCoef))-1)
        probes_in_data <- c(length(intersect(rownames(betas),names(horvathCoef))),
                 length(intersect(rownames(betas),names(hannumCoef))),
                 length(intersect(rownames(betas),names(skinbloodCoef))),
                 length(intersect(rownames(betas),names(phenoageCoef))),
                 length(intersect(rownames(betas),names(telomereCoef))),
                 length(intersect(rownames(betas),yangCoef)),
                 length(intersect(rownames(betas),names(ZhangCoef))),
                 length(intersect(rownames(betas),
                 unlist(DunedinPoAm38::getRequiredProbes()$DunedinPoAm_38))),
                 length(intersect(rownames(betas),names(linCoef))),
                 length(intersect(rownames(betas),names(weidnerCoef))),
```

```
                length(intersect(rownames(betas),names(vidalbraloCoef))))
  probe_df <- data.frame(biomarker, probes_in_biomarker, probes_in_data)
  probe_df$Percentage_Coverage   <- round((probe_df$probes_in_data/probe_df$probes_in_biomarker)*100,2)
  return(probe_df)}
```

2.4 agep_NG Function:

Function to calculate DNAm epigenetics biomarkers except proprietary GrimAge, GrimAge2, and their components.

```
trafo <- function(x,adult.age=20) { x=(x+1)/(1+adult.age); y=ifelse(x<=1, log( x),x-1);y}
anti.trafo <- function(x,adult.age=20) { ifelse(x<0, (1+adult.age)*exp(x)-1,
                                              (1+adult.age)*x+adult.age) }
agep_NG <- function(betas, coeff = NULL, method = c('horvath', 'hannum','skinblood',
                 'phenoage', 'telomere', 'yang','zhang', 'lin', 'weidner', 'vidalbralo'),...){
if(method == 'horvath'){
        ages <- as.matrix(apply(betas,2,function(x){
        miss <- names(coeff)[-1]%in%names(na.omit(x))
        coef2 <- coeff[-1][miss]
        data <- x[names(coef2)]
        pre <- data %*% coef2 + coeff[1]
        anti.trafo(pre,  adult.age=20)}))}
if(method == 'hannum'){
        ages <- as.matrix(apply(betas,2,function(x){
        miss <- names(coeff)%in%names(na.omit(x))
        coef2 <- coeff[miss]
       data <- x[names(coef2)]
       data %*% coef2 + 0}))}
if(method=='skinblood'){
        ages <- as.matrix(apply(betas,2,function(x){
        miss <- names(coeff)%in%names(na.omit(x)) coef2
        <- coeff[miss]
        data <- x[names(coef2)]
        pre <- data %*% coef2 + coeff[1]
        anti.trafo(pre,  adult.age=20)}))}
if(method == 'phenoage'){
        ages <- as.matrix(apply(betas,2,function(x){
        miss <- names(coeff)[-1]%in%names(na.omit(x))
        coef2 <- coeff[-1][miss]
        data <- x[names(coef2)]
        data %*% coef2 + coeff[1]}))}
if(method == 'telomere'){
        ages <- as.matrix(apply(betas,2,function(x){
        miss <- names(coeff)[-1]%in%names(na.omit(x))
        coef2 <- coeff[-1][miss]
        data <- x[names(coef2)]
        data <- x[names(coef2)]
        data %*% coef2 + coeff[1]}))}
if(method == 'yang'){
        common.v <- intersect(rownames(betas),coeff)
        map.idx <- match(common.v,rownames(betas))
        ages <- colMeans(betas[map.idx,])}
if(method == 'zhang'){
        ages <- as.matrix(apply(betas,2,function(x){
```

```
        miss <- names(coeff)[-1]%in%names(na.omit(x))
        coef2 <- coeff[-1][miss]
        data <- x[names(coef2)]
        data %*% coef2 + coeff[1]})))}
if(method == 'lin'){
        ages <- as.matrix(apply(betas,2,function(x){ miss
        <- names(coeff)[-1]%in%names(na.omit(x)) coef2 <-
        coeff[-1][miss]
        data <- x[names(coef2)]
        data %*% coef2 + coeff[1]})))}
if(method == 'weidner'){
        ages <- as.matrix(apply(betas,2,function(x){ miss
        <- names(coeff)[-1]%in%names(na.omit(x)) coef2 <-
        coeff[-1][miss]
        data <- x[names(coef2)] data %*% coef2 + coeff[1]})))}
if(method == 'vidalbralo'){
        ages <- as.matrix(apply(betas,2,function(x){ miss
        <- names(coeff)[-1]%in%names(na.omit(x)) coef2 <-
        coeff[-1][miss]
        data <- x[names(coef2)] data %*% coef2 + coeff[1]})))}
  return(ages)}
```

3 Creating Methylation Objects

Code to read in iDats were to create an RGChannelSet Extended object to use for pre-processing and normalization.

```
sampleInfo <- read.csv("NHANES_Samplesheet.csv", header = T, row.names = 1, skip = 7)
sampleInfo$Basename <- paste(sampleInfo$Sentrix_ID, sampleInfo$Sentrix_Position, sep="_")
sampleInfo$Chip_Position <- sampleInfo$Basename
sampleInfo$Basename <- file.path("iDats", sampleInfo$Basename)
NHANES_RG_Raw <- read.metharray(basenames = sampleInfo$Basename, extended = T)
identical(sampleNames(NHANES_RG_Raw), sampleInfo$Chip_Position)
sampleNames(NHANES_RG_Raw) <- sampleInfo$Sample_Group identical(rownames(sampleInfo),
sampleNames(NHANES_RG_Raw)) save(NHANES_RG_Raw, file = "NHANES_RG_Raw.RData")
```

4 Outlier Detection & Removal

4.1 Quality Control Check

Code to analyze the control probes to see how the array performed and to see if there were any outlier samples present.

```
MSet.raw <- preprocessRaw(NHANES_RG_Raw) qc.raw <-
getQC(MSet.raw)
MSet.raw.QC <- addQC(MSet.raw, qc.raw) plotQC(qc.raw,
badSampleCutoff = 10.5) NHANES_qcraw <-
as.data.frame(qc.raw@listData) NHANES_qcraw$Sample_ID <-
qc.raw@rownames
NHANES_qcraw$Threshold <- (NHANES_qcraw$mMed + NHANES_qcraw$uMed)/2 outliers
<- NHANES_qcraw$Sample_ID[NHANES_qcraw$Threshold <= 10.5]
save(MSet.raw, file = "MSet.raw.RData")
save(NHANES_qcraw, file = "NHANES_qcraw.RData")
```

4.2 Removing Outliers

```
NHANES_RG_OR <- NHANES_RG_Raw[,!sampleNames(NHANES_RG_Raw) %in% outliers]
save(NHANES_RG_OR, file = "NHANES_RG_OR.RData")
```

5 Background Subtraction & Color Correction

Function from the minfi package used to remove background signal and control for dye bias of the array.

```
NHANES_RG_BC <- preprocessNoob(NHANES_RG_OR) save(NHANES_RG_BC,
file = "NHANES_RG_BC.RData")
```

6 General Imputation and Normalization

Code used for a subset of the epigenetic biomarkers (Lin, Weidner, Vidal-Bralo, Yang, Zhang, DunedinPoAm) that used beta-mixture quantile normalization (BMIQ).

```
NHANES_RG_BC_BMIQ <- BMIQ(NHANES_RG_BC, nfit = 100000) # This produces just a matrix
save(NHANES_RG_BC_BMIQ, file = "NHANES_RG_BC_BMIQ.RData")
```

7 Horvath Imputation and Normalization

Imputation and normalization of Horvath, Hannum, SkinBlood, GrimAge, GrimAge2, and PhenoAge.

7.1 Data Preparation

```
dat <- as.data.frame(getBeta(NHANES_RG_BC)) input=
cbind(rownames(dat), dat)
colnames(input)[1]="ProbeID"
ann=read.csv("datMiniAnnotation3.csv")
check=is.element(ann$Name,input$ProbeID)
miss.cpg=ann$Name[!check]
input.subject=colnames(input)[-1]
nsubject=dim(input)[2]-1
nmiss.cpg=length(miss.cpg)
add=data.frame(matrix(data=NA,nrow=nmiss.cpg,ncol=dim(input)[2]))
add[1:5,1:5]
names(add)=names(input) add[,1]=miss.cpg
betas_horvath_new=rbind(input,add)
betas_horvath_new=betas_horvath_new[betas_horvath_new$ProbeID %in% ann$Name,]
```

7.2 Imputation and Normalization

```
source("Horvath_Norm.R")
gold=read.csv("datMiniAnnotation3_Gold.csv")
datMethUsed= t(betas_horvath_new[,-1]) colnames(datMethUsed)=as.character(betas_horvath_new[,1])
input.na=apply(is.na(datMethUsed),2,sum)
input.na=data.frame(var=names(input.na),nmiss=input.na)
input.na=subset(input.na,nmiss>0)
var.miss=input.na$var
ck=is.element(var.miss,gold$CpG)
table(ck)
datMethUsedGI = datMethUsed
for(k in 1:length(var.miss)){
gold0=subset(gold,CpG==var.miss[k])
index=is.na(datMethUsedGI[,var.miss[k]])
datMethUsedGI[index,var.miss[k]]=gold0$gold}
temp=apply(is.na(datMethUsedGI),2,sum)
summary(temp) betas_horvath_N=BMIQcalibration(datM=datMethUsedGI,goldstandard.beta=gold$gold)
NHANES_Beta_Horvath <- as.data.frame(betas_horvath_N)
save(NHANES_Beta_Horvath, file = "NHANES_Beta_Horvath.RData")
```

8 Epigenetic Age Prediction

8.1 DNAm Epigenetic Biomarker Missing Probes

Code for calculation of missing probes (except for GrimAge, GrimAge2, and their components).

```
betas <- getBeta(NHANES_RG_BC)
NHANES_Biomarker_Probe_Count <- DNAm_Biomarker_Probe_Count_NG(betas)
write.csv(NHANES_Biomarker_Probe_Count, file = "NHANES_Biomarker_Probe_Count.csv")
save(NHANES_Biomarker_Probe_Count, file = "NHANES_Biomarker_Probe_Count.RData")
```

8.2 DNAm Biomarker Calculation

```
betasH <- NHANES_Beta_Horvath
betasBMIQ <- NHANES_RG_BC_BMIQ
NHANES_DNAm_Meta <- as.data.frame(agep_NG(betasH, method = "horvath", coeff=horvathCoef))
colnames(NHANES_DNAm_Meta)[1] <- "Horvath"
NHANES_DNAm_Meta$Hannum <- agep_NG(betasH, method = "hannum", coeff = hannumCoef)
NHANES_DNAm_Meta$SkinBlood <- agep_NG(betasH, method = "skinblood", coeff = skinbloodCoef)
NHANES_DNAm_Meta$PhenoAge <- agep_NG(betasH, method = "phenoage", coeff = phenoageCoef)
NHANES_DNAm_Meta$DNAmTL <- agep_NG(betasBMIQ, method = "telomere", coeff = telomereCoef)
NHANES_DNAm_Meta$Yang <- agep_NG(betasBMIQ, method = "yang", coeff = yangCoef)
NHANES_DNAm_Meta$Zhang <- agep_NG(betasBMIQ, method = "zhang", coeff = ZhangCoef)
NHANES_DNAm_Meta$Lin <- agep_NG(betasBMIQ, method = "lin", coeff = linCoef)
NHANES_DNAm_Meta$Weidner <- agep_NG(betasBMIQ, method = "weidner", coeff = weidnerCoef)
NHANES_DNAm_Meta$VidalBralo <- agep_NG(betasBMIQ, method="vidalbralo", coeff=vidalbraloCoef)
DunedinPoAm <- as.data.frame(DunedinPoAm38::PoAmProjector(betasBMIQ))
NHANES_DNAm_Meta$DunedinPoAm <- DunedinPoAm$DunedinPoAm_38
NHANES_DNAm_Meta$SP_ID <- rownames(NHANES_DNAm_Meta)
save(NHANES_DNAm_Meta, file = "NHANES_DNAm_Meta.RData")
```

8.3 GrimAge, GrimAge2, and Components

This code is proprietary so cannot be shared. We received permission from the authors to use the code for calculation of GrimAge. GrimAge2, and their components for this instance only. Code was deleted after the calculation was performed.

9 Cell Type Prediction

Cell type prediction (CD8+ T-cells, CD4+ T-cells, natural killer cells, B-cells, monocytes, neutrophils) was done using the EPIC blood reference dataset and the IDOL probes[27].

```
data("IDOLOptimizedCpGs") load("Data/FlowSorted.Blood.EPIC_ref.RData")
NHANES_CTPs <- estimateCellCounts2(NHANES_RG_Raw, compositeCellType = "Blood", probeSelect =
                    "IDOL", referencePlatform = "IlluminaHumanMethylationEPIC",
                    IDOLOptimizedCpGs=IDOLOptimizedCpGs, referenceset="FlowSorted.Blood.EPIC_ref")
NHANES_CTPs <- as.data.frame(NHANES_CTPs$counts)
NHANES_CTPs$SP_ID <- rownames(NHANES_CTPs)
NHANES_DNAm_Meta <- join(NHANES_DNAm_Meta, NHANES_CTPs, by = "SP_ID")
save(NHANES_DNAm_Meta, file="NHANES_DNAm_Meta.RData")
```

10 Sample Mismatch Analysis

10.1 Epigenetic Age Deviation

Identifying samples where chronological age was more than 2 standard deviations away from

predicted age using five epigenetic predictors.

```
NHANES_DNAm$clock_deviated_2sd <- 0
for (clock in c("Hannum","BloodSkin","Zhang","DNAmTIMP1","GrimAge")){
NHANES_DNAm$temp <- NHANES_DNAm[,clock] - NHANES_DNAm$RIDAGEYR
NHANES_DNAm$deviated_2sd[abs(NHANES_DNAm$temp - mean(NHANES_DNAm$temp,na.rm=T))>
  sd(NHANES_DNAm$temp,na.rm=T)*2 & !is.na(NHANES_DNAm$temp)] <-
NHANES_DNAm$deviated_2sd +1}
```

10.2 Cell Count Comparison

Identifying samples where cell counts were more than 2 standard deviations away from predicted cell proportions.

```
NHANES_DNAm$celltype_mo <- NHANES_DNAm$LBXMOPCT - NHANES_DNAm$Mono*100
NHANES_DNAm$celltype_ne <- NHANES_DNAm$LBXNEPCT -
NHANES_DNAm$Neu*100NHANES_DNAm$celltype_deviated_2sd <- 0
NHANES_DNAm$celltype_deviated_2sd[abs(NHANES_DNAm$celltype_mo)
        >sd(NHANES_DNAm$celltype_mo,na.rm=T)*2 &
                !is.na(NHANES_DNAm$celltype_mo)] <- NHANES_DNAm$celltype_deviated_2sd +1
NHANES_DNAm$celltype_deviated_2sd[abs(NHANES_DNAm$celltype_ne)
        >sd(NHANES_DNAm$celltype_ne,na.rm=T)*2 &
                !is.na(NHANES_DNAm$celltype_ne)] <- NHANES_DNAm$celltype_deviated_2sd +1
```

10.3 XY Chromosome Comparison

Identifying any samples which don't have the expected chromosomal ploidy.

```
NHANES_DNAm$XY_Estimation <- getSex(NHANES_MS)
NHANES_DNAm$mismatch <- abs(NHANES_DNAm$XY_Estimation-NHANES_DNAm$RIAGENDR)
```

10.4 Sample Mismatch Assessment

Identifying any samples mismatched on two or more of the three criteria.

```
table(paste0(NHANES_DNAm$clock_deviated_2sd,
NHANES_DNAm$celltype_deviated_2sd,NHANES_DNAm$mismatch))
```

References:

1. Bird, A. DNA methylation patterns and epigenetic memory. *Genes Dev.* **16**, 6–21 (2002).
2. Henikoff, S. & Greally, J. M. Epigenetics, cellular memory and gene regulation. *Current Biology* **26**, R644–R648 (2016).
3. Horvath, S. & Raj, K. DNA methylation-based biomarkers and the epigenetic clock theory of ageing. *Nat Rev Genet* **19**, 371–384 (2018).
4. Horvath, S. *et al.* Accelerated epigenetic aging in Down syndrome. *Aging Cell* **14**, 491–495 (2015).
5. Fransquet, P. D., Wrigglesworth, J., Woods, R. L., Ernst, M. E. & Ryan, J. The epigenetic clock as a predictor of disease and mortality risk: a systematic review and meta-analysis. *Clinical Epigenetics* **11**, 62 (2019).
6. Marioni, R. E. *et al.* The epigenetic clock is correlated with physical and cognitive fitness in the Lothian Birth Cohort 1936. *International Journal of Epidemiology* **44**, 1388–1396 (2015).
7. Wagner, W. Epigenetic aging clocks in mice and men. *Genome Biol* **18**, 1–3 (2017).
8. McEwen, L. M., Goodman, S. J., Kobor, M. S. & Jones, M. J. The DNA Methylome: An Interface Between the Environment, Immunity, and Ageing. in *The Ageing Immune System and Health* (eds. Bueno, V., Lord, J. M. & Jackson, T. A.) 35–52 (Springer International Publishing, Cham, 2017). doi:10.1007/978-3-319-43365-3_3.
9. RStudio Team. RStudio: Integrated Development Environment for R. RStudio, PBC. (2023).
10. R Core Team. R: A Language and Environment for Statistical Computing. R Foundation for Statistical Computing (2023).
11. Horvath, S. DNA methylation age of human tissues and cell types. *Genome Biol* **14**, 1–20 (2013).
12. Hannum, G. *et al.* Genome-wide Methylation Profiles Reveal Quantitative Views of Human Aging Rates. *Mol Cell* **49**, 359–367 (2013).
13. Horvath, S. *et al.* Epigenetic clock for skin and blood cells applied to Hutchinson Gilford Progeria Syndrome and ex vivo studies. *Aging (Albany NY)* **10**, 1758–1775 (2018).
14. Lu, A. T. *et al.* DNA methylation GrimAge strongly predicts lifespan and healthspan. *Aging (Albany NY)* **11**, 303–327 (2019).
15. Lu, A. T. *et al.* DNA methylation GrimAge version 2. *Aging* **14**, 9484–9549 (2022).
16. Levine, M. E. *et al.* An epigenetic biomarker of aging for lifespan and healthspan. *Aging (Albany NY)* **10**, 573–591 (2018).
17. Lu, A. T. *et al.* DNA methylation-based estimator of telomere length. *Aging (Albany NY)* **11**, 5895–5923 (2019).
18. Yang, Z. *et al.* Correlation of an epigenetic mitotic clock with cancer risk. *Genome Biol* **17**, 1–18 (2016).
19. Zhang, Q. *et al.* Improved precision of epigenetic clock estimates across tissues and its implication for biological ageing. *Genome Medicine* **11**, 54 (2019).
20. Lin, Q. *et al.* DNA methylation levels at individual age-associated CpG sites can be indicative for life expectancy. *Aging (Albany NY)* **8**, 394–401 (2016).
21. Weidner, C. I. *et al.* Aging of blood can be tracked by DNA methylation changes at just three CpG sites. *Genome Biol* **15**, 1–12 (2014).
22. Vidal-Bralo, L., Lopez-Golan, Y. & Gonzalez, A. Simplified Assay for Epigenetic Age Estimation in Whole Blood of Adults. *Frontiers in Genetics* **7**, (2016).

23. Belsky, D. W. *et al.* Quantification of the pace of biological aging in humans through a blood test, the DunedinPoAm DNA methylation algorithm. *eLife* **9**, e54870 (2020).
24. Teschendorff, A. E. *et al.* A beta-mixture quantile normalization method for correcting probe design bias in Illumina Infinium 450 k DNA methylation data. *Bioinformatics* **29**, 189–196 (2013).
25. Houseman, E. A. *et al.* DNA methylation arrays as surrogate measures of cell mixture distribution. *BMC Bioinformatics* **13**, 86 (2012).
26. Koestler, D. C. *et al.* Blood-based profiles of DNA methylation predict the underlying distribution of cell types. *Epigenetics* **8**, 816–826 (2013).
27. Salas, L. A. *et al.* An optimized library for reference-based deconvolution of whole-blood biospecimens assayed using the Illumina HumanMethylationEPIC BeadArray. *Genome Biol* **19**, 1–14 (2018).
28. Koestler, D. C. *et al.* Improving cell mixture deconvolution by identifying optimal DNA methylation libraries (IDOL). *BMC Bioinformatics* **17**, 120 (2016).
29. Fortin, J.-P. *et al.* Functional normalization of 450k methylation array data improves replication in large cancer studies. *Genome Biol* **15**, 1–17 (2014).
30. Du, P., Kibbe, W. A. & Lin, S. M. lumi : a pipeline for processing Illumina microarray. *Bioinformatics* **24**, 1547–1548 (2008).
31. Pidsley, R. *et al.* Critical evaluation of the Illumina MethylationEPIC BeadChip microarray for whole-genome DNA methylation profiling. *Genome Biol* **17**, 1–17 (2016).
32. Pidsley, R. *et al.* A data-driven approach to preprocessing Illumina 450K methylation array data. *BMC Genomics* **14**, 293 (2013).
33. Price, E. M. *et al.* Additional annotation enhances potential for biologically-relevant analysis of the Illumina Infinium HumanMethylation450 BeadChip array. *Epigenetics & Chromatin* **6**, 4 (2013).
34. Fortin, J.-P., Triche, T. J. & Hansen, K. D. Preprocessing, normalization and integration of the Illumina HumanMethylationEPIC array with minfi. *Bioinformatics* **33**, 558–560 (2017).
35. Johnson, W. E., Li, C. & Rabinovic, A. Adjusting batch effects in microarray expression data using empirical Bayes methods. *Biostatistics* **8**, 118–127 (2007).
36. Benjamini, Y. & Yekutieli, D. False Discovery Rate–Adjusted Multiple Confidence Intervals for Selected Parameters. *Journal of the American Statistical Association* **100**, 71–81 (2005).
