# Supplementary material for: Exploring the role of β2-microglobulin in the relationship between physical activity and DNAm-predicted PhenoAge: Evidence from a population-based and mice single-cell RNA-sequencing study
Source: J Adv Res. 2025 Nov 22;86:743–55. doi: 10.1016/j.jare.2025.11.047 (PMC13453862; doi:10.1016/j.jare.2025.11.047)
Supplement: Supplementary file 2 [file mmc1.docx]

**Table S1.** Characteristics of participants in this study.

| Table for Categorical Variables | | |
| --- | --- | --- |
| Variable | Category | Number and Percentage |
| Gender | Male | 474 (50.64%) |
|  | Female | 462 (49.36%) |
| Race/Ethnicity | Non-Hispanic White | 791 (84.54%) |
|  | Non-Hispanic Black | 51 (5.48%) |
|  | Mexican American | 26 (2.74%) |
|  | Other Race/Ethnicity | 68 (7.23%) |
| Marital Status | Never married | 25 (2.65%) |
|  | Married/living with partner | 696 (74.33%) |
|  | Widowed/divorced | 215 (23.01%) |
| Education | Below high school | 47 (4.98%) |
|  | High school | 342 (36.58%) |
|  | College or above | 547 (58.44%) |
| Poverty Income Ratio | < 1 | 54 (5.80%) |
|  | [1,3) | 287 (30.67%) |
|  | ≥ 3 | 595 (63.54%) |
| Body Mass Index (kg/m²) | < 25 | 282 (30.16%) |
|  | [25,30) | 364 (38.94%) |
|  | ≥ 30 | 289 (30.90%) |
| Smokers | Never smoker | 404 (43.19%) |
|  | Former smoker | 423 (45.23%) |
|  | Current smoker | 108 (11.58%) |
| Alcohol Drinkers | Nondrinker | 304 (32.49%) |
|  | Moderate alcohol use | 562 (60.06%) |
|  | High alcohol use | 70 (7.45%) |
| Table for Continuous Variables | | |
| Variable | Mean ± SE | |
| Age | 62.54 ± 0.38 | |
| Physical Activity (MET per week) | 7,352.52 ± 460.81 | |
| β2M | 1,682,136.33 ± 6,798.92 | |
| GDF15 | 911.43 ± 4.82 | |
| CystatinC | 598,043.12 ± 1,550.54 | |
| TIMP1 | 34,872.32 ± 59.25 | |
| ADM | 346.05 ± 1.27 | |
| PAI1 | 17,187.49 ± 114.62 | |
| Leptin | 9,262.90 ± 159.35 | |
| PACKYRS | 16.73 ± 0.56 | |
| CRP | 0.64 ± 0.02 | |
| logA1C | 1.72 ± 0.001 | |
| GrimAge | 62.76 ± 0.31 | |
| GrimAge2 | 68.25 ± 0.32 | |

Notes: Number (percentage) for category variables and Mean ± SE for continuous variables. Abbreviations:β2-Microglobulin (β2M); Growth Differentiation Factor 15 (GDF15); Tissue Inhibitor of Metalloproteinases 1 (TIMP1); Adrenomedullin (ADM); Plasminogen Activator Inhibitor 1 (PAI1); Pack Years of Smoking (PACKYRS); C-Reactive Protein (CRP); Log-Transformed Hemoglobin A1c (logA1C).

**Supplement Figure 1.** Quality control, feature selection, and UMAP clustering of single-cell RNA-seq data across experimental groups.

(A, B) Violin plots showing the distribution of three quality metrics: nFeature_RNA (detected genes per cell), nCount_RNA (total RNA count per cell), and percent.mt (mitochondrial RNA percentage) for Old Control (OC, red), Old Exercise (OE, green), Young Control (YC, cyan), and Young Exercise (YE, purple). YE demonstrates the highest transcriptional diversity, while OC shows reduced diversity.

(C) Scatter plots highlighting highly variable genes (red dots), such as Hbb-bs and S100a8, critical for downstream clustering.

(D) UMAP projection of single-cell RNA-seq data. Different colors represent the experimental groups, and distinct clusters indicate transcriptionally unique cell populations, with YE and YC groups showing greater immune diversity and activity compared to OC.

**Supplementary Table 2.** Associations between physical activity and Log-based DNAm derived epigenetic biomarkers.

|  |  |  | Fully Adjusted Model | |
| --- | --- | --- | --- | --- |
|  |  |  | β (95% CI) | *p-value* |
| Log (GDF15) |  |  | 0.001(-0.014, 0.017) | 0.846 |
| Log (CystatinC) |  |  | 0.004(-0.008, 0.000) | 0.030 |
| Log (TIMP1) |  |  | -0.003(-0.006, 0.001) | 0.093 |
| Log (ADM) |  |  | -0.002(-0.006, 0.001) | 0.205 |
| Log (PAI1) |  |  | 0.003(-0.007, 0.012) | 0.550 |
| Log (Leptin) |  |  | 0.022(-0.004,0.048) | 0.089 |
| Log (PACKYRS) |  |  | -0.038(-0.123, 0.047) | 0.355 |
| Log (CRP) |  |  | -0.02(-0.064,0.023) | 0.330 |
| Log (A1C) |  |  | 0(-0.002,0.003) | 0.733 |
| Log (GrimAge) |  |  | -0.007(-0.016, 0.002) | 0.125 |
| Log (GrimAge2) |  |  | -0.006(-0.014, 0.001) | 0.096 |

Notes: Fully Adjusted Model: gender, race, marital status, education, poverty status, body mass index, smoking status, and alcohol use status were adjusted. CI, confidence interval. The results show the percentage change in the Log-based DNAm derived epigenetic biomarkers per 100 MET-minutes/week increase in physical activity, expressed as % change (95% CI: lower bound, upper bound).
